# The Degradation of Absorbable Surgical Threads in Body Fluids: Insights from Infrared Spectroscopy Studies

**DOI:** 10.3390/ijms252011333

**Published:** 2024-10-21

**Authors:** Katarzyna Merkel, Katarzyna Grzybowska, Aleksandra Strach, Marcin Gierek

**Affiliations:** 1 Institute of Materials Engineering, Faculty of Science and Technology, University of Silesia, 75 Pułku Piechoty 1a, 41-500 Chorzów, Poland; 2 Institute of Physics, Faculty of Science and Technology, University of Silesia, 75 Pułku Piechoty 1a, 41-500 Chorzów, Poland; katarzyna.grzybowska@us.edu.pl; 3 Doctoral School, University of Silesia, Bankowa 14, 40-032 Katowice, Poland; aleksandra.strach@us.edu.pl; 4 Center for Burns Treatment im. Dr Sakiel, ul. Jana Pawła II 2, 41-100 Siemianowice Śląskie, Poland

**Keywords:** surgical threads, hydrolytic degradation, tensile strength, SEM, FTIR, PCA analysis, poliglecaprone 25, poliglactin-910, polydioxanone, triclosan, PDS, PDS Plus, Vicryl, Vicryl Plus, Monocryl Plus

## Abstract

This study investigates the degradation of six different types of absorbable surgical threads commonly used in clinical practice, focusing on their response to exposure to physiological fluids. The threads were subjected to hydrolytic and enzymatic degradation in physiological saline, bile, and pancreatic juice. Our findings demonstrate that bile and pancreatic juice, particularly when contaminated with bacterial strains such as *Escherichia coli*, *Klebsiella* spp., and *Enterococcus faecalis*, significantly accelerate the degradation process. Using Fourier-transform infrared spectroscopy (FTIR), scanning electron microscopy (SEM), and tensile strength testing, we observed distinct differences in the chemical structure and mechanical integrity of the sutures. Principal component analysis (PCA) of the FTIR spectra revealed that PDS threads exhibited the highest resistance to degradation, maintaining their mechanical properties for a longer duration compared with Monocryl and Vicryl. These results highlight the critical role of thread selection in gastrointestinal surgeries, where prolonged exposure to bile and pancreatic juice can compromise the suture integrity and lead to postoperative complications. The insights gained from this study will contribute to improving the selection and application of absorbable threads in clinical settings.

## 1. Introduction

Synthetic polymeric biomaterials are widely used across various medical fields, particularly in surgery, due to their unique properties, including biocompatibility, elasticity, sterility, and mechanical strength. These materials are subject to degradation processes that are influenced by the pH of physiological fluids and by the duration of contact between the polymeric materials and biological tissues [1]. Both synthetic and natural polymers have been extensively used in surgical procedures, necessitating a comprehensive evaluation when selecting suture materials, particularly due to the specificity of wound healing processes [2]. Surgical threads must possess essential attributes such as biocompatibility, elasticity, sterility, adequate mechanical strength, and the capability of forming secure knots [3,4,5]. Understanding the physical and mechanical properties of surgical threads, such as tensile strength, stiffness, and suture dimensions, is critical. Surgical threads are generally classified into three main categories: monofilament, multifilament (braided or twisted), and coated varieties [6,7].

The first biodegradable polymers used extensively in surgical sutures were poly(lactic acid) (PLA) and poly(glycolic acid) (PGA) [8,9,10]. Common absorbable thread materials include catgut, polylactide, polyglycolide, and polyglycolide–polylactide copolymers. In contrast, non-absorbable threads are made from materials such as polyester, polypropylene, polyamide, and silk [11,12]. While synthetic biopolymers based on ester compounds can undergo biodegradation facilitated by bacterial and fungal activity, they exhibit prolonged resistance to biodegradation in animal and human organisms due to the absence of the requisite enzymes [13,14]. Despite this, these materials retain bioresorbable qualities, although the process is considerably slow and typically begins with hydrolytic degradation [15,16]. Polymer degradation involves the loss of physical and chemical properties through processes such as photodegradation, hydrolysis, and oxidation, often catalyzed by microorganisms, leading to a reduction in molecular weight [17]. The chemical structure of polymers, particularly the presence of hydrolytic and oxidative bonds, is the most important factor influencing degradation [18]. Polymer chain hydrolysis may occur via chemical or enzymatic pathways, which are affected by internal factors such as composition, molecular geometry, crystallinity, and hydrophilicity [19], as well as by external factors like pH, temperature, and enzyme presence or concentration [20].

Degradation of biomaterials is essential for biocompatibility, since changes in physicochemical properties during degradation can affect both functionality and the biological response. A comprehensive understanding of biomaterial degradation factors is pivotal for the development of degradable polymer-based systems and products [21]. When exposed to body fluids, materials can undergo significant changes due to chemical, physical, mechanical, and biological interactions with their environment. Recent research has primarily focused on degradation mechanisms in polymers derived from poly(lactic acid) (PLA) and copolymers of lactide and glycolide (PLGA) [21,22,23,24,25,26,27,28,29,30,31,32,33]. Previous studies have mainly examined the effect of buffered physiological saline solution (PBS) and controlled pH variations in PBS on biopolymer degradation [14,26,29,34,35].

This study focuses on evaluating the physicochemical properties of various surgical threads used in gastrointestinal anastomoses, such as jejuno–jejunal, jejuno–biliary, and jejuno–pancreatic anastomoses. In the early stages of healing, the integrity of these anastomoses is highly dependent on the sutures used. The selection of suitable threads during pancreatic surgery is crucial to the success of the procedure [36,37]. Each thread type displays different mechanical properties and tissue responses. Ideally, the material should be non-traumatic, induce minimal inflammation, and absorb at a suitable rate to prevent anastomotic breakdown in cases of delayed healing [38,39,40,41]. Gastrointestinal anastomoses are particularly vulnerable to the effects of bile and pancreatic juice, which accelerate the degradation of surgical materials. There is considerable evidence of premature suture dehiscence in gastrointestinal anastomoses exposed to bile and pancreatic juice, which often complicates the healing process [37,38,39,40,41]. Despite this, both clinical and experimental data on the performance of threads in pancreatic and biliary duct anastomoses remain limited [42,43,44,45,46,47,48,49]. Clinically, it is often challenging to determine whether the suture material has degraded or remains intact when an anastomotic leak occurs, as this can only be evaluated during re-laparotomy if the site is accessible [50,51,52].

Fourier-transform infrared (FTIR) spectroscopy is a valuable tool for assessing changes in the chemical bonds of biopolymers, such as carbonyl, hydroxyl, and ether groups [53,54,55]. Data from FTIR spectra support the study of alterations in the mechanical strength of surgical threads, which serve as primary indicators of polymer degradation [56,57,58]. This study aims to investigate the processes that occur when commonly used absorbable suture materials—such as polydioxanone (PDS), polyglactin-910 (Vicryl), and poliglecaprone 25—are exposed to bile, pancreatic juice, and infected environments. These polymers are frequently utilized in general surgery and are often exposed to these fluids. Understanding their biodegradation mechanisms will help improve suture selection for specific anastomoses and enhance the success of surgical procedures.

## 2. Results and Discussion

### 2.1. Experimental and Simulated Spectra of Surgical Thread Biopolymer Band Assignments

In this section, we present the FTIR spectra of the raw (initial) surgical threads, which will serve as reference samples. The threads under investigation are composed of aliphatic polyesters containing one or more of the four fundamental building blocks: glycolide, L-lactide, p-dioxanone, and ε-caprolactone. All polymers constructed from these building blocks are semi-crystalline [10]. FTIR spectra are highly sensitive to the amorphous and crystalline nature of samples and can, thus, be used to monitor the development of the crystalline phase during the degradation process [52]. Glycolide and L-lactide are considered “hard” segments due to their rigidity and reduced flexibility, whereas ε-caprolactone and p-dioxanone are considered “soft” segments, which provide more flexibility to the suture fibers. The fastest in vivo absorption rates are exhibited by absorbable threads composed entirely of glycolide. Those made entirely of L-lactide or p-dioxanone have the most extended absorption times [10]. The spectra of the polymers under study are complex and rich in bands, which presents a challenge for analysis. Accordingly, theoretical FTIR spectra were simulated using the B3LYP/6-311G(d,p) method [59,60] (details are described in Section 3.2.3) for isolated molecules consisting of only two monomer units.

**Figure 1:**
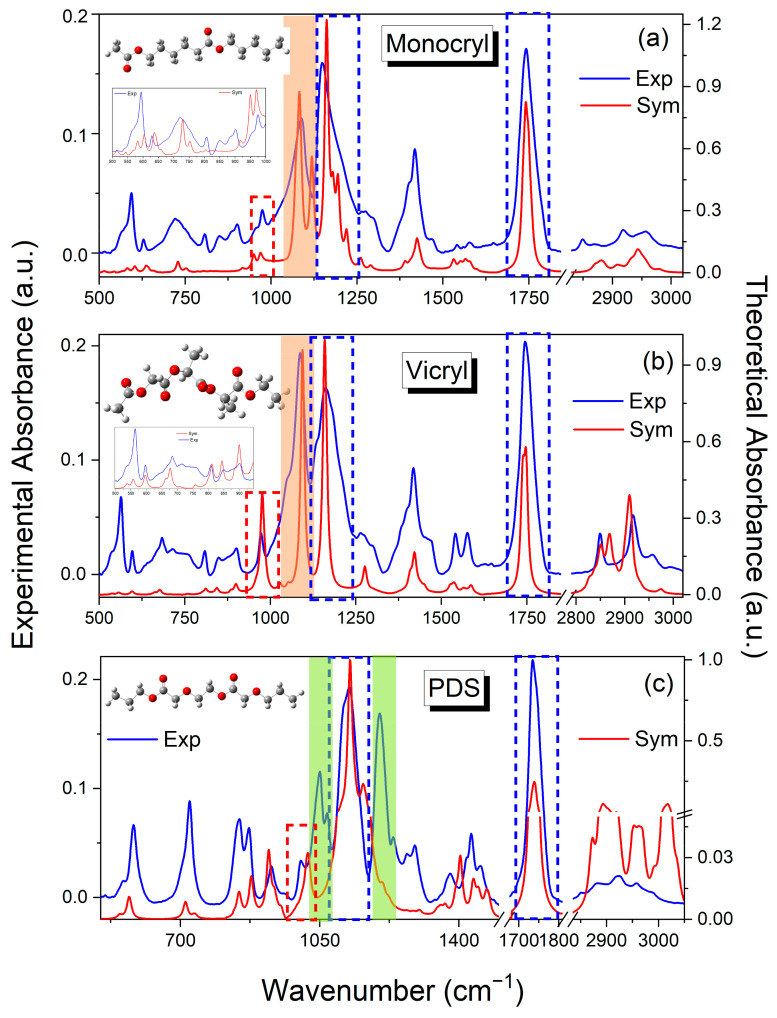
The comparison of the experimental spectra with the theoretical one (B3-LYP/6-311G (d,p)) for the synthetic absorbable threads in the region of 500–3000 cm^−1^. (**a**) Poly(glycolide/ε-caprolactone) copolymer (Monocryl); (**b**) Poly(glycolide/L-lactide) copolymer (Vicryl); (**c**) Poly-p-dioxanone. The blue dashed line shows the ester group, and red represents the ether group. Orange and green highlights show the characteristic bands for glycolide and dioxanone building blocks, respectively.

Figure 1 presents a comparison of the simulated FTIR spectra of the polymers with the spectrum of the raw threads. In the spectra, characteristic bands for the corresponding building blocks are marked with colors. It can be observed that the simulated spectra very accurately reproduce the prominent characteristic bands for ester groups. In contrast, the most remarkable differences are observed for Vicryl and Monocryl in the range from 500 to 900 cm^−1^. These differences are attributable to the limited number of monomer units included in the simulated spectrum and the absence of consideration of intermolecular interactions.

Similarly, minor discrepancies are observed in the ester band range of the PDS spectrum. The experimental spectrum of the studied polyesters can be divided into three main spectral regions: (1) 500–900 cm^−1^; (2) 1000–1800 cm^−1^; (3) 2800–3000 cm^−1^. The initial range (500–900 cm^−1^) is primarily concerned with the bending vibrations of carbon (C-C) and hydrogen (C-H) atoms in methylene and methyl groups (CH_2_ and CH_3_), as well as the out-of-plane bending vibrations of the carbonyl group (γ C=O) and the in-plane bending vibration of the carbonyl group (β C=O) observed in the wavenumber range of 500–650 cm^−1^. The second range is a typical fingerprint region, including characteristic stretching vibrations of the C-O-C group in ethers (1050, 1060, 1080 cm^−1^) and esters at wavenumbers from 1150–1270 cm^−1^. At approximately 1750 cm^−1^, a pronounced peak is observed, corresponding to the stretching vibration of the carbonyl group in esters (ν C=O).

This range is most sensitive to changes resulting from hydrolysis and oxidation reactions. The last range (2800–2950 cm^−1^) includes stretching vibrations of C-H in methylene and methyl groups (ν CH_2_, ν CH_3_). Furthermore, the spectrum exhibits characteristic bands situated at approximately 1415–1430 cm^−1^, which are indicative of bending vibrations of C-H in methylene groups (β CH_2_, scissor), in addition to bands belonging to skeletal C-C-C vibrations within the range of 800–1200 cm^−1^. The Supplementary Materials include Tables S1–S3, which provide a comparative analysis of the wavenumbers and relative intensities of all bands in the experimental and theoretical spectra for all polymers. Based on the theoretical spectra and a review of the literature, detailed assignments of bands to the vibrations of corresponding functional groups were made. Table 1 provides a general summary of the wavenumbers at which the most characteristic bands in the initial polymer spectra are observed and their assignments. Figure S1 presents a comparison of the spectra for raw surgical threads and their coated counterparts. The observed differences are minimal and primarily manifest as alterations in the intensity of the bands.

### 2.2. Principal Component Analysis

**Figure 2:**
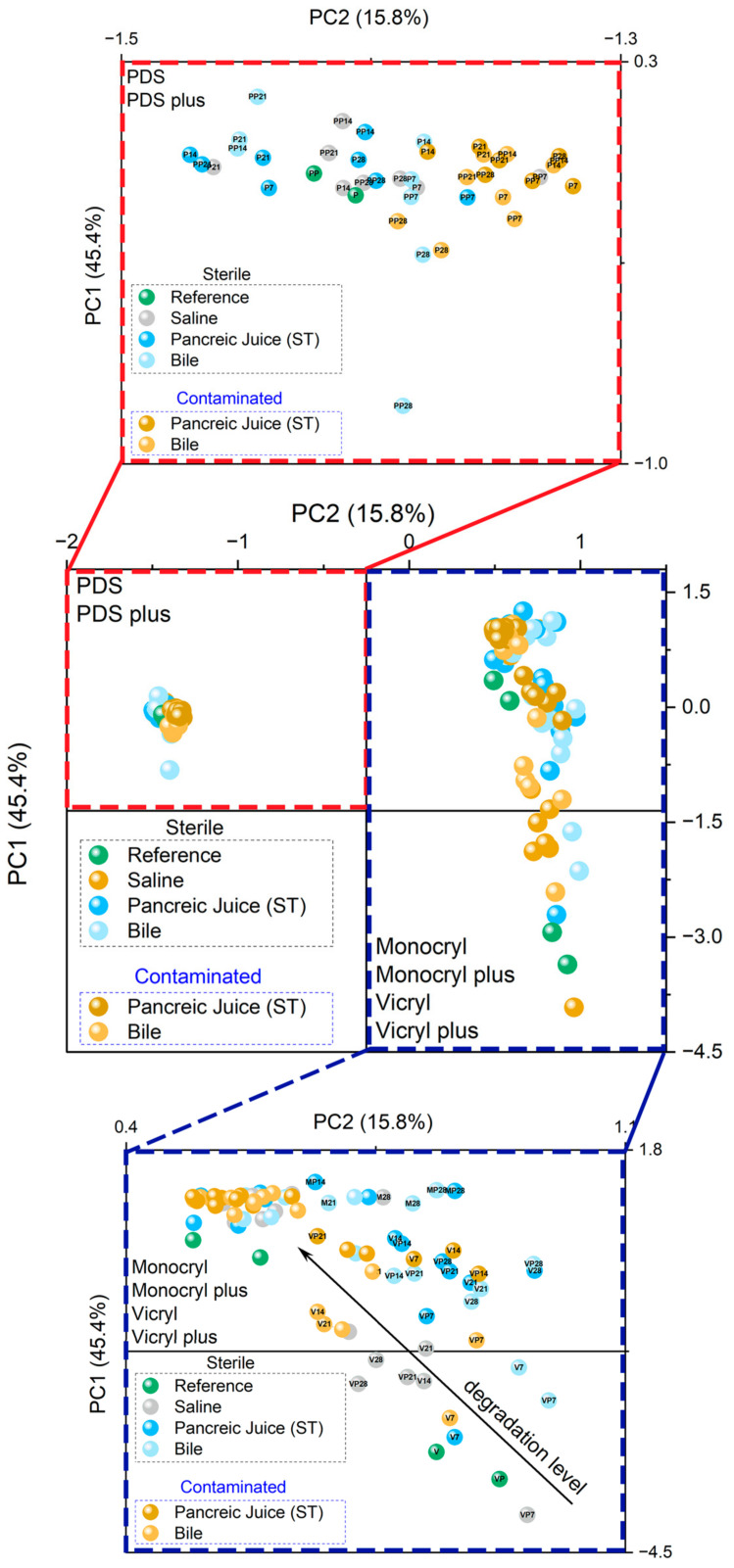
Principal component analysis (PCA) of the vector-normalized second-derivative FTIR spectra from the 950–1800 cm^−1^ region. The analysis encompasses data for various polymers across different environments and time points. Initially, PCA was conducted on the entire dataset, which was subsequently subdivided into two distinct panels to more clearly illustrate the differences between samples. The red-dashed rectangle highlights data specific to PDS and PDS Plus, while the blue-dashed rectangle encompasses data for Monocryl, Monocryl Plus, Vicryl, and Vicryl Plus.

The infrared spectroscopy data comparing the spectra of different polymers across various environments and time intervals revealed distinct differences in the second derivatives within the fingerprint region of 950–1800 cm^−1^. These observations suggest a significant impact of environmental conditions and exposure time on the structural modifications of the polymer matrices. To further investigate these effects, principal component analysis (PCA) was performed on individually prepared data matrices. The data were categorized based on the environment (sterile, contaminated, saline, pancreatic juice, bile), utilizing a correlation matrix and focusing on the two principal components, PC1 and PC2 (Figure 2).

According to the PCA results, the cluster separation along the PC1 axis explains nearly 50% of the variance, indicating that the infrared spectra of PDS and PDS plus form distinct groups relative to Monocryl, Monocryl Plus, Vicryl, and Vicryl Plus. Additionally, the lower percentage contribution of PC2 differentiates Monocryl, Monocryl plus, Vicryl, and Vicryl plus. In contrast, samples within the PDS and PDS plus clusters remain in close proximity to each other. These data clearly highlight a statistical difference between the polymer groups, with an insignificant impact of the environment and time observed for PDS and PDS plus, which contrasts with significant differences in the other polymers.

A more detailed analysis of the sub-group data reveals minor differences between the infrared spectra in sterile and contaminated environments, as explained by PC1. Considering only a bile solution, PC2 further illustrates that some spectral differences for PDS plus emerge only after 28 days. This suggests that only structural changes occur under these specific conditions.

On the other hand, more pronounced differences are evident in the second group of polymers, with PC2 distinguishing samples based on polymer type, environment, and exposure time. Monocryl and Vicryl are separately grouped, pointing to the statistically distinct behavior of these polymers. When exposed to prolonged environmental conditions such as saline, pancreatic juice, and bile (reference or seven days), the spectra of Vicryl and Vicryl plus exhibit very similar spectral patterns. Furthermore, the spectra of Vicryl and Vicryl Plus in a sterile saline environment show minor alterations compared with those in other environments, where more prolonged exposure leads to more significant modifications in the infrared spectra due to more vigorous structural alterations. Additionally, samples in contaminated environments undergo faster spectral changes compared with similar samples in sterile conditions, highlighting the more significant impact of such environments on polymer alterations.

Lastly, the infrared spectra of Monocryl and Monocryl plus, irrespective of the environment and time, exhibit close similarity to each other, suggesting a robust environmental impact independent of exposure time. One plausible explanation is the rapid degradation of these polymers under all tested conditions, especially when compared with Vicryl and Vicryl plus, which demonstrate much better stability—specifically, up to 14 days in sterile pancreatic juice and bile, up to 28 days in saline, and only up to 7 days in contaminated solutions (Figure 2). However, to confirm the observed effects and to explain more precisely the environmental and time effects, further analyses were performed and summarized in latter parts of the paper.

### 2.3. Mechanisms and Spectral Analysis of Surgical Thread Degradation

Biomedical polymers degrade through four main mechanisms: hydrolysis (reaction with water in tissues), oxidation (due to oxidants produced by tissues), enzymatic degradation, and physical degradation (e.g., water swelling, mechanical loading, and wear) [16,29,30,31,55,61]. The hydrolytic degradation of polymers involves the breaking of ester bonds. Initially, water molecules interact with the polymer surface by forming hydrogen bonds with hydrophilic groups, which is followed by bond hydrolysis. Hydrolysis reactions can be catalyzed by acids, bases, salts, or enzymes [62]. Typically, homogeneous hydrolytic degradation of ester biopolymers can occur via bulk erosion, surface erosion, or a combination of both mechanisms [14,29]. Bulk erosion is defined as the process whereby water diffusion occurs at a faster rate than the degradation of the material, resulting in uniform degradation and subsequent mass loss throughout the material’s volume. In contrast, surface erosion is characterized by degradation primarily at the material’s surface.

Another degradation mechanism is oxidation, which involves the action of peroxides produced by the body. In contrast to polyolefins, polyesters are less prone to the generation of free radicals, which makes them less susceptible to oxidation. This is supported by the findings of studies such as those conducted by [16,29,61]. Nevertheless, hydrolysis reactions can result in the formation of hydroxyl groups at the chain ends, which can then be oxidized to aldehyde or ketone groups.

#### 2.3.1. Sterile Environment

**Figure 3:**
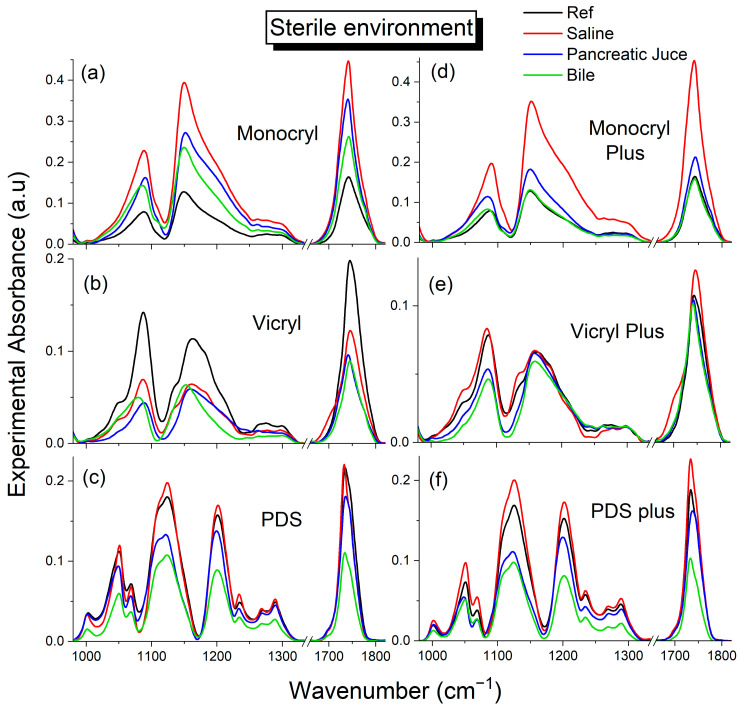
FTIR spectra of the fingerprint range of the hydrolytic bonds (ester and ether groups) for absorbable threads immersed in the sterile body fluids for 21 days. (**a**) Monocryl; (**b**) Vicryl; (**c**) PDS; (**d**) Monocryl Plus; (**e**) Vicryl Plus; (**f**) PDS plus. *Black solid line*—raw threads (before degradation); *red line*—saline solution; *blue line*—pancreatic juice; *green line*—bile.

The Supplementary Materials include Figures S2–S4, which compare the FTIR spectra of all the studied surgical threads. These figures illustrate the evolution of polymer structure changes when exposed to body fluids (saline, pancreatic juice, and bile) under sterile conditions over 7, 14, and 21 days. The spectral region most sensitive to degradation processes in polyesters includes vibrations from ester and ether groups, which is referred to as the fingerprint region due to its characteristic representation of the material. Figure 3 presents a comparison of suture spectra in the fingerprint region before degradation (raw threads) and after 21 days of degradation under sterile conditions. In the poly(glycolide/ε-caprolactone) copolymer (Monocryl), a significant increase in the intensity of the ester group (COO-) bands is observed (Figure 3a). This increase is related to the attack of water on the polymer’s hydrophilic centers, leading to chain plasticization through the formation of hydrogen bond networks. The most pronounced effect is seen in Monocryl threads exposed to saline solution. Significant broadening of bands around 1150 cm^−1^ and 1200 cm^−1^ corresponds to the asymmetric stretching vibration of the C-O group (ν_as_ C-C(O)-O). The band at 1150 cm^−1^ is attributed to mixed crystalline and amorphous domains, while the peak around 1200 cm^−1^ is associated with crystalline regions [63,64,65,66]. As water is absorbed, the polymer chains become more flexible, potentially increasing the crystalline phase, as indicated by the increased intensity of the 1200 cm^−1^ band.

For the poly(glycolide/L-lactide) copolymer (Vicryl) (Figure 3b), a significant decrease in the intensity of all ester bands sensitive to hydrolysis is observed, indicating an advanced degradation process due to ester bond hydrolysis. Compared with Monocryl, Vicryl displays more bands characteristic of the amorphous phase. The 1047 cm^−1^ band, associated with the stretching vibration of C-O in the C-O-C(CH_3_) group, and the 1133 cm^−1^ band, characteristic of the stretching vibration of the C-C(O)-O group in the L-lactide segment, both disappear due to hydrolytic degradation in pancreatic juice and bile solutions. It confirms that degradation initially occurs in the amorphous regions. The degradation of ester bonds results in the formation of low-molecular-weight products, which, in turn, decreases the amorphous phase and increases the crystalline phase in the Vicryl structure (see the increase in the intensity of the band at 1200 cm^−1^).

In the case of polydioxanone (PDS) threads (Figure 3c), a decrease in the intensity of bands associated with the stretching vibration of the C-C(O)-O group in esters is observed, although the changes are less pronounced compared with Vicryl threads. The band associated with the ether bridge (ν_as_ C-O-C) exhibited the least pronounced changes in intensity, with the most notable observations occurring in bile solution, where broadening and a shift of the maximum toward higher frequencies were observed. The hydrolysis profiles of triclosan-coated threads exhibited similar patterns, though the rate of changes under sterile conditions was significantly slower.

In order to determine the dynamics of the structural changes in response to external factors, the percentage decrease/increase in integral absorbance for selected bands with respect to the raw threads before degradation was determined using the following equation:

(1) $$∆X=\frac{X_{ref\;}-X_{i\;}}{X_{ref\;}}\;\cdot 100\%$$

where Δ*X* denotes either the percentage change in band absorbance (A) or the percentage change in mechanical strength (σ) post-degradation in comparison to the reference thread strength. The findings are presented in Figures S5–S7 in the Supplementary Materials. For all polymers, bands related to the asymmetric stretching vibration in the ester groups (1150–1250 cm^−1^; ν_as_ C-C(O)-O) and the asymmetric stretching vibration of the ether bridge (1000–1100 cm^−1^; ν_as_ O-C-C) were analyzed. Additionally, the distinctive bands characteristic of alkyl chains were evaluated for Monocryl and Vicryl. These encompassed the deformation vibration of methylene groups (~1415 cm^−1^; β_s_ CH_2_, scissor) and the symmetric stretching vibration of the methyl group (~2920 cm^−1^; ν_s_ CH_3_).

In the case of Monocryl, for all bands, an increase in absorbance relative to the pre-degradation reference was observed, resulting in a diminished percentage change (Figure S11). Notably, in a sterile environment, this phenomenon was most pronounced for the saline solution, exhibiting an approximately 200% change after 21 days. This increase in absorbance is due to the plasticizing effect of the absorbed water and is evidence of volumetric erosion. As a consequence of plasticization, polymer chains exhibit enhanced mobility, which correlates with increased crystallinity, as evidenced by a considerable increase in the integral absorbance of the deformation vibrations of the methylene group (~1418 cm^−1^). The formation of hydrogen bond networks with hydrophilic groups in the polymer increased the absorbance, with the maximum effect observed for asymmetric stretching vibrations in the ester group (ν_as_ C-C(O)-O) in saline solution. In contrast, the smallest increase in absorbance, approximately 60%, was observed for the bile solution in a sterile medium.

For the Vicryl threads, all the bands exhibited a decrease in absorbance relative to the reference, resulting in an increased percentage change (Figure S12). Following 21 days of incubation in all the body fluids, comparable levels of structural alterations were observed in both sterile and infected conditions. The observed percentage decrease in absorbance for ester group vibrations, around 100%, is indicative of the second stage of degradation, namely ester bond breakdown due to hydrolysis. In contrast, a converse trend was noted for skeletal vibrations involving the methyl group deformation vibration (νCCC + δCH_3_), which is known to be sensitive to polymer crystallinity. The literature indicates that bands at 1415, 975, and 902 cm^−1^ correspond to crystalline domains or blocks of glycolide in the polymer [63,64,65,66]. The growth integral absorbance of the band with a peak at 975 cm^−1^ is attributed to heightened crystallinity. As degradation progresses, the cleavage of polymer chains facilitates the higher ordering of the shorter chains, leading to enhanced crystallinity. The intensive chain degradation was observed in infected environments for both pancreatic juice and bile. In a sterile medium, the degradation process was slightly accelerated in the bile medium.

Regarding the polydioxanone (PDS) threads, a significant percentage decrease in absorbance was observed after 21 days for the band (~1050 cm^−1^) associated with the stretching vibrations of the ether bridge, characteristic of the amorphous phase.

#### 2.3.2. Bacterially Contaminated Environment

**Figure 4:**
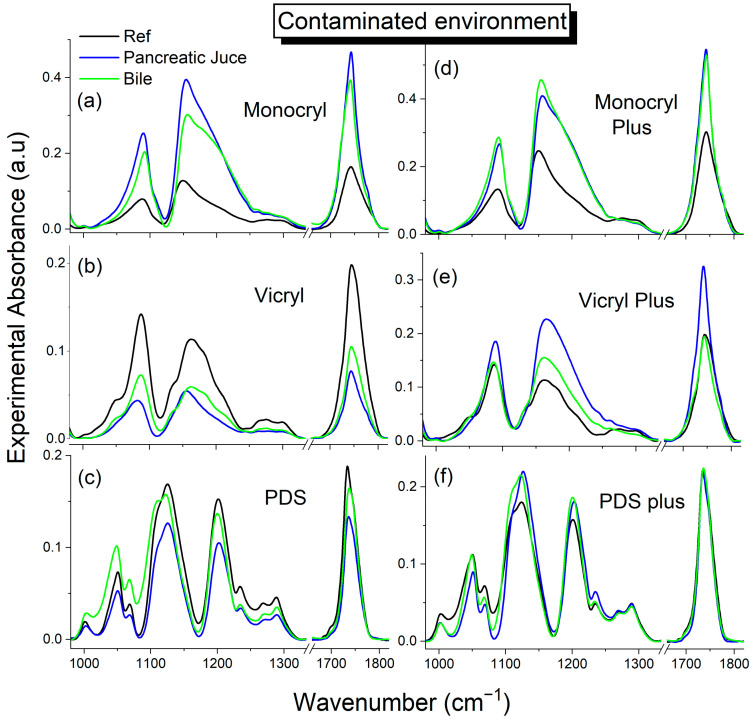
FTIR spectra of characteristic ester bands sensitive to degradation under bacterial contamination after 21 days. (**a**) Monocryl; (**b**) Vicryl; (**c**) PDS; (**d**) Monocryl Plus; (**e**) Vicryl Plus; (**f**) PDS plus. *Black solid line*—raw thread (before degradation); *blue line*—pancreatic juice; *green line*—bile.

Figures S8–S13 summarize the evolution of polymer structure changes when exposed to body fluids under infected conditions over 7, 14, and 21 days. The contaminated material was obtained by infecting sterile pancreatic juice and sterile bile with strains of bacteria. Figure 4 presents a comparison of suture spectra in the fingerprint region before degradation (raw threads) and after 21 days of degradation under infected conditions. The spectral changes observed for all polymers in an infected environment are similar to those in a sterile environment. However, for PDS surgical threads, the hydrolysis process is significantly slighter compared with the other polymers. In the PDS suture spectrum exposed to pancreatic juice, there is a notable decline in the intensity of the 1050 cm^−1^ band, characteristic of the ether bridge, as well as in bands in the 1150–1200 cm^−1^ range associated with ester groups, observed in comparison with the bile solution. This indicates a more pronounced extent of enzymatic hydrolysis in the infected environment. Conversely, the rate of change for coated threads is relatively slower. In the spectra of all coated threads, an increase in the intensity of ester group bands is observed, indicating the formation of hydrogen bonds and the subsequent plasticization of the polymer chains.

#### 2.3.3. Identification of Degradation Products

The degradation process occurs through a series of erosive mechanisms occurring in multiple stages, which result in the reversion of the polymer back into its constituent monomers. Initially, water diffuses into the amorphous regions of the polymer matrix, facilitating the breaking of ester bonds. Subsequently, a hydrolytic attack targets crystalline regions, and, ultimately, the collapse of crystalline regions leads to the dissolution of the polymer chain. It is notable that certain bands within the spectral range of 900–1300 cm^−1^, corresponding to stretching vibrations of ether and ester groups, and within the range of 1700–1800 cm^−1^, which are associated with stretching vibrations of the carbonyl group, may not be easily visible. The initial spectrum analysis can reveal ambiguous features such as convex inflection points or gradient changes along the band contour. The determination of the positions of overlapping bands, particularly when broadened (1000–1200 cm^−1^), presents further challenges. Next, the second derivative of the absorbance function with respect to wavenumber is computed to enhance the precision of the analysis and to identify potential hydrolytic degradation products.

**Figure 5:**
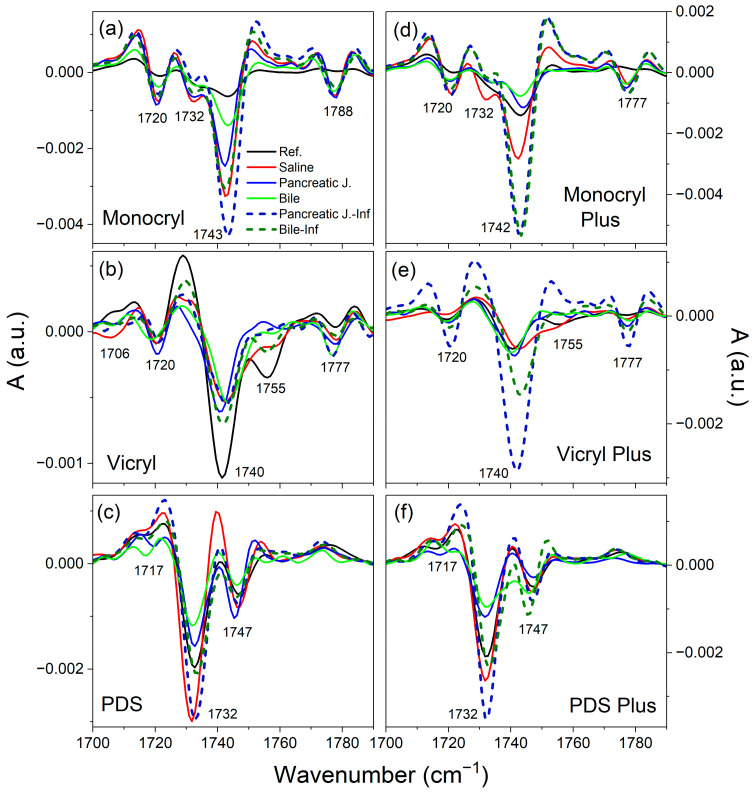
Position of bands on the spectra of raw polymers and after 21 days of incubation in body fluids within the range of 1700–1800 cm^−1^ (stretching vibration of the C=O group), determined on the basis of second derivatives. Smoothing was performed using a Savitsky–Golay filter with a score of 11. (**a**–**c**) Uncoated surgical threads; (**d**–**f**) Triclosan-coated threads. Results for both sterile and contaminated environments.

The minima observed in the second derivative of the absorbance function correspond to maxima or convex inflection points in the spectrum, providing additional insights into band positions, including those obscured in the original spectrum. Moreover, the second derivative yields bands with areas under the curve proportional to those in the original spectrum, thereby elucidating the band intensities. Figure 5 illustrates the second derivatives, smoothed uniformly (Savitsky–Golay; 11 points) across the carbonyl group stretching vibration range (1700–1800 cm^−1^) for both reference and degraded spectra following 21 days of suture immersion in various media. This spectral region is recognized in the literature as conducive to assessing polyester crystallinity, which is a critical determinant of degradation kinetics due to preferential hydrolysis and oxidation or enzymatic attack within amorphous domains.

Tables S1–S3 present the assignment of spectral bands and their association with crystalline or amorphous phases. In the second derivative spectra for Monocryl (Figure 5a,d), in addition to the dominant carbonyl group maximum at 1743 cm^−1^, characteristic of semi-crystalline polymers, less intense bands at 1720, 1732, and 1788 cm^−1^ are observed, which are absent in the reference spectrum. These bands correspond to high-crystalline, low-crystalline, and intermolecular interaction phases, respectively. The latter is most pronounced in saline solution. Similarly, Vicryl thread spectra (Figure 5b,e) exhibit a primary band at 1740 cm^−1^, with additional bands indicating increased crystallinity after degradation at 1777 and 1720 cm^−1^. Notably, the amorphous phase band at 1755 cm^−1^ disappears post-degradation, except in bile-infected environments. Additionally, a low-intensity band in the 1705–1710 cm^−1^ range, varying with the medium, is linked to hydrolytic degradation products, likely α-hydroxy acids. Degradation-induced pH changes may trigger inflammatory responses.

The raw PDS thread (Figure 5c,f) exhibits intense maxima at 1732 and 1747 cm^−1^, representing crystalline and amorphous phases, respectively, with a low-intensity band at approximately 1720 cm^−1^. In contrast to Vicryl threads, the disappearance of the amorphous phase band at 1747 cm^−1^ is incomplete. The band at 1720 cm^−1^ shifts based on the medium, indicating a likely association with a carbonyl group and hydrogen bond. It is consistent with the presence of the carbonyl group prior to degradation. Hydrolytic degradation effects are most pronounced in the Vicryl thread carbonyl group stretching band region, as revealed by the second derivative analysis.

The analysis of the bands of ester and ether groups (Figure 3) in a physiological environment (saline, pancreatic juice, and bile) indicates that polymers are degraded primarily under the influence of water molecules. The degradation process can be divided into three stages: (1) The action of water molecules primarily occurs in the amorphous regions, forming a network of hydrogen bonds, which causes the plasticization of the polymer chains (an increase in the intensity of the bands of ester groups). (2) The ester bond of the chain breaks, and water molecules begin to penetrate the polymer matrix, resulting in a decrease in the intensity of the ester bands and the production of liquid-soluble oligomers (Figure 3b,c,e,f and Figure 4b,c). (3) In the third stage, the oligomers are gradually hydrolyzed to monomeric acids under the catalysis of biological enzymes. This step was only slightly visible for the Vicryl threads, and the appearance of a new, low-intensity band indicative of a small number of free carboxyl groups was observed (Figure 5b; 1705–1710 cm^−1^). In subsequent steps, the monomers would be metabolized to water and carbon dioxide. This step was not observed during the 28-day experiment. The deterioration of these materials over the experiment is associated with only the initial two stages. The second stage involves three main types of mechanisms, namely catalytic degradation under the influence of acids in bile solution and enzymatic degradation in pancreatic juice, random chain cleavage, and surface or volumetric degradation. Random fission of ester bonds was fastest in bile solution in a sterile medium, while it was slowest in infected medium in pancreatic juice. The coating threads with triclosan in the infected environment unquestionably decelerated the stage of ester bond rupture. In contrast with sterile bile and pancreatic juice, we observed a reversal of the trend and an increase in the intensity of ester bands.

### 2.4. Mechanical Strength Evaluation of Surgical Threads

The mechanical strength of surgical threads is crucial to their effectiveness in resisting knots and tensions when joining soft tissues. Surgical threads with insufficient strength are susceptible to intraoperative or, more importantly, post-operative damage. Tensile strength, meaning the maximum force threads can withstand before breaking, is a fundamental property that is assessed in the study of such materials.

Control time points for strength measurements were set at regular intervals (every 7 days) until the 28th day of the investigation. It is typical for tissue healing to span between the seventh and fourteenth days; thus, control assessments were scheduled accordingly. Some researchers extend control periods beyond 14 days; for instance, Freudenberg et al. extended measurements up to the 21st day of suture immersion in biological fluids [34]. The study ended on the 28th day of exposure to biological fluids due to the observation of in vivo complications stemming from anastomotic dehiscence after 14 days of surgery [67]. Anastomotic dehiscence typically manifests between the fifth and eighth post-operative days [68]. Mast et al. and Martens et al. have reported that the strength of small bowel anastomosis returns to baseline after four weeks of healing, while the large intestine achieves 75% of its original strength after four months [69,70]. Deveney et al. observed the dehiscence of Vicryl threads on the 21st–22nd day of the study [71]. This suggests that bowel anastomosis healing extends beyond 7–14 days, justifying the duration of in vitro studies. Details regarding the strength testing methodology and comprehensive statistical analyses have been outlined in a previous publication [36].

**Figure 6:**
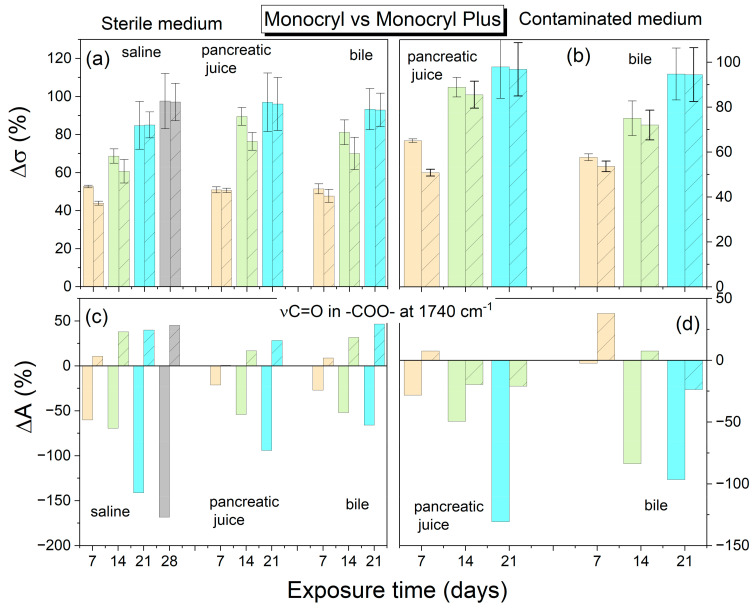
Average percentage change in tensile strength and ester band absorbance for Monocryl/Monocryl plus threads relative to the reference (raw suture before degradation). (**a**,**c**) Sterile environment; (**b**,**d**) Contaminated environment. *Top figures*—strength results; *bottom figures*—FTIR results (ν C=O vibration). *Light orange*—7 days; *light green*—14 days; *light cyan*—21 days. The absorbance error is ±2 cm^−1^. Patterned columns represent coated sutures.

**Figure 7:**
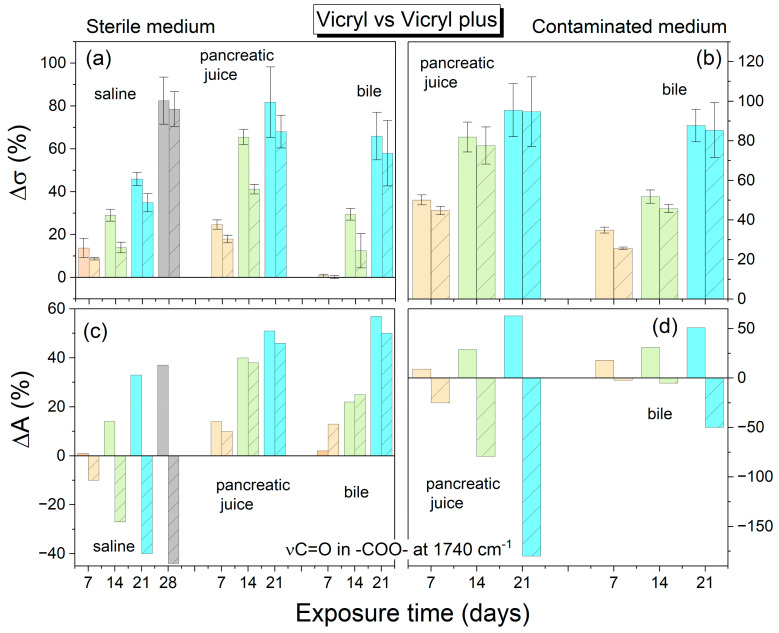
Average percentage change in tensile strength and ester band absorbance for Vicryl/Vicryl plus threads relative to the reference (raw suture before degradation). (**a**,**c**) Sterile environment; (**b**,**d**) Contaminated environment. *Top figures*—strength results; *bottom figures*—FTIR results (ν C=O vibration). *Light orange*—7 days; *light green*—14 days; *light cyan*—21 days; *light gray*—28 days. The absorbance error is ±2 cm^−1^. Patterned columns represent coated sutures.

**Figure 8:**
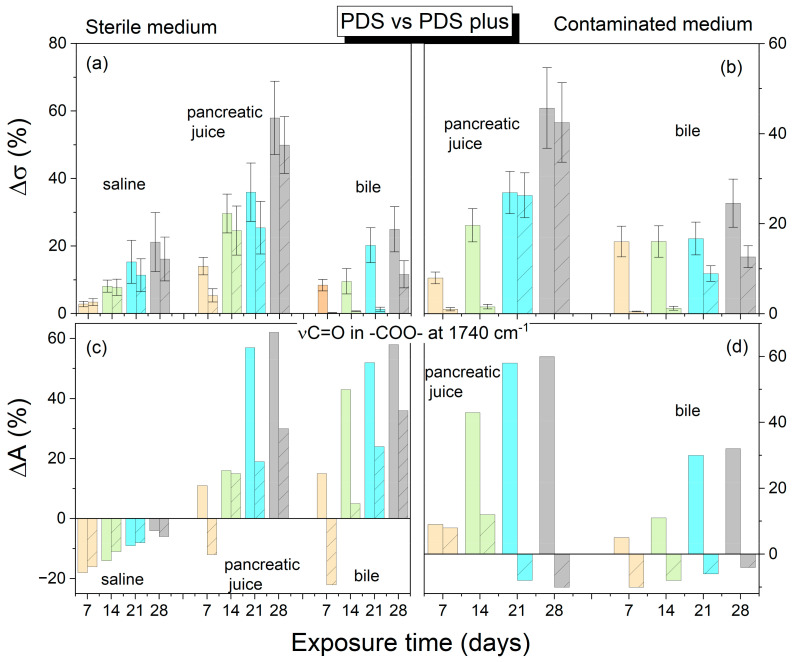
Average percentage change in tensile strength and ester band absorbance for PDS/PDS plus threads relative to the reference (raw suture before degradation). (**a**,**c**) Sterile environment; (**b**,**d**) Contaminated environment. *Top figures*—strength results; *bottom figures*—FTIR results (ν C=O vibration). *Light orange*—7 days; *light green*—14 days; *light cyan*—21 days; *light gray*—28 days. The absorbance error is ±2 cm^−1^. Patterned columns represent coated sutures.

This study found PDS to be the most stable suture, exhibiting the most negligible percentage decrease in strength by the 28th day, irrespective of the environment. Among the investigated threads, only PDS retained its integrity throughout the study period. In order to compare the strength test results with the structural changes of the polymer, the percentage decrease in mechanical strength compared to the initial strength of the references was calculated according to the defined formula in Equation (1). In addition, the percentage change in the integral absorbance for the stretching vibration band of the C=O group at 1750 cm^−1^ was also calculated. Figure 6, Figure 7 and Figure 8 illustrate a comparison of the percentage change in tensile strength among the reference threads (Monocryl, Vicryl, PDS) and those coated with an antibacterial layer (Monocryl Plus, Vicryl Plus, and PDS Plus) subjected to various environments. The initial average tensile strengths for uncoated threads were 236.5, 293, and 117.2 MPa (Monocryl, Vicryl, PDS, respectively), while those for coated threads were slightly higher at 268.7, 282.8, and 127.8 MPa (Monocryl Plus, Vicryl Plus, PDS Plus). The Vicryl threads exhibited the highest initial tensile strength. All the surgical threads showed a significant decrease in tensile strength after 14 days of exposure to body fluids, which is consistent with the results of the FTIR analysis. Monocryl threads (Figure 6) demonstrated a loss of approximately 90% of their tensile strength and around 80% for coated threads in pancreatic juice after 14 days in sterile conditions. The most significant increase in C=O band absorbance (70%) was observed in physiological saline solution, which can be attributed to water absorption and plasticization. In pancreatic juice and bile, hydration competes with direct ester bond hydrolysis, resulting in a minor absorbance increase (approximately 50% after 14 days).

The Vicryl threads lose approximately 66% of their tensile strength in the pancreatic juice in sterile environments after 14 days, as demonstrated in Figure 7. This is in contrast with a 30% loss observed in bile.

The lowest tensile strength decrease was observed for the PDS threads after 14 days in sterile conditions. The most significant decrease was observed in pancreatic juice (30%) and bile (20%), which correlated with respective decreases in absorbance of 36% and 13%. With regard to the effects of bile and pancreatic juice, the PDS strands showed the most extended periods of stability (with no significant decrease in tensile strength), namely 14 and 21 days in the sterile and infected biliary environment, respectively (Figure 8). Only small decreases in intensity associated with ester bond degradation were observed.

Both hydration of the polymers through hydrogen bond formation, leading to plasticization, and direct hydrolysis of the bonds contributed equally to the reduction in mechanical strength observed in the polymers tested.

In comparison with sterile conditions, the contaminated pancreatic juice and bile resulted in a reduction in thread strength, with the exception of PDS Plus threads. Throughout the study, the decline in strength of the PDS Plus threads remained consistent across both sterile and infected environments.

The application of a triclosan coating to surgical threads leads to a marginal decrease in strength. Furthermore, regarding alterations in the FTIR spectrum, the application of an antibacterial substance to threads retards the chemical hydrolysis reaction in infected environments.

### 2.5. Morphological Evaluation of Surgical Threads

**Figure 9:**
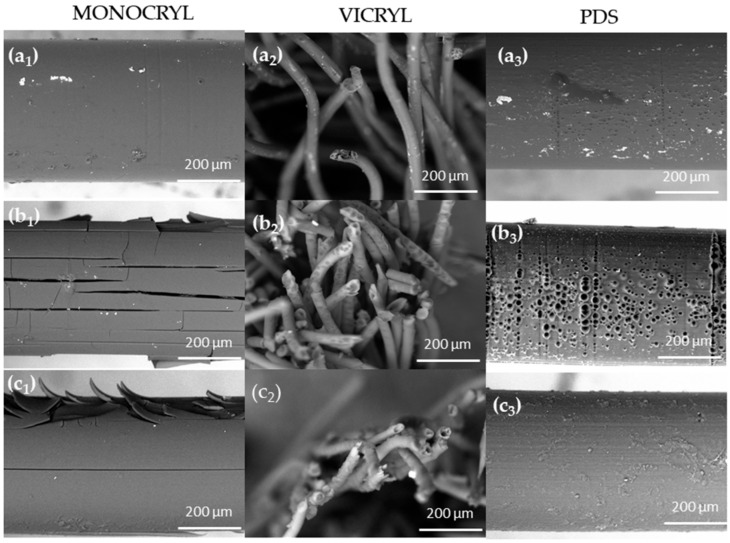
Scanning electron micrographs of uncoated threads immersed for 21 days in sterile saline, pancreatic, and bile juices. (**a1**–**a3**) Saline; (**b1**–**b3**) pancreatic juice; (**c1**–**c3**) bile; *Subscripts:* 1- Monocryl, 2 -Vicryl, 3 -PDS threads.

The surface of the surgical suture samples was analyzed using scanning electron microscopy (SEM). Details of the measurement methodology were described in a previous study [36]. The SEM results obtained for uncoated threads exposed to physiological saline, pancreatic juice, and bile under sterile conditions after 21 days are shown in Figure 9. The images show a homogeneous structure without microorganisms and impurities. External factors influencing the degree of degradation include moisture, enzymes, pH, and microorganisms. In the Supplementary Materials, Figures S14 and S15 present the surface changes on the threads in an infected environment. No disintegration was observed on the surface of the PDS threads, which was consistent with the results of the infrared analysis. The observation of the deformation vibration of the methylene groups (CH_2_) at a wavenumber of approximately 1420 cm^−1^ revealed that no disappearance of the band was observed in the PDS spectra, in contrast to the observations made in the Vicryl and the Monocryl threads (at 1450 and 1430 cm^−1^). The bands in the range of 1430–1450 cm^−1^ correspond to the deformation vibrations of methylene groups in the alkyl chain in the amorphous region of polymer fibers. This indicates that after 21 days, PDS still has polymer fibers rather than the oligomers or monomers seen in the Vicryl and the Monocryl threads.

Furthermore, the Monocryl threads exposed to pancreatic juice showed significantly more cracking and surface disintegration compared with those exposed to bile. The results indicate that a slowdown in degradation can be observed in bile, where the pH was approximately 6.8, in comparison with pancreatic juice with its higher pH. The pH effect on the degradation profiles is also associated with the disruption of hydrogen bond interactions in an alkaline environment [71]. As crystalline domains are more resistant to water molecule diffusion, hydrolysis is likely to occur along the longitudinal and transverse directions of the fibers associated with amorphous interlamellar regions. The morphological changes that occur during the degradation process are dependent on the dominant direction and the relative distribution of crystalline blocks in neighboring microfibrils. When crystalline domains are distributed at different levels, lateral diffusion is hindered, resulting in the formation of longitudinal cracks (Figure 9). This phenomenon, which represents the initial stage of degradation, is particularly prevalent in Monocryl. Microcracks that form in later stages of degradation will continue to hydrolyze along the cross-sectional plane, where less resistant amorphous regions are located. It can be observed that after 21 days in both sterile and infected environments, surface erosion is more likely for both the Monocryl and the PDS threads. This is undoubtedly related to the higher degree of crystallinity and hydrophobicity compared with the Vicryl threads. Enzymatic hydrolysis of the PDS strands and the Monocryl threads in pancreatic juice occurs mainly at the polymer surface, as it is difficult for the hydrophilic enzyme to diffuse into the hydrophobic polymer.

In the case of the Vicryl threads, after 21 days of the experiment, apparent unraveling of multifilament surgical threads and distinct volumetric erosion were observed (Figure 9). The presence of voids within the fiber, which had been exposed to both pancreatic juice and bile, was clearly observed. It can be stated that, in the case of Vicryl strands in a pancreatic juice solution, both surface and volume degradation can be observed. Conversely, hydrolysis in a bile environment primarily occurs through a volume erosion mechanism. This is undoubtedly associated with the much higher hydrophilicity and lower degree of crystallinity of the polymer [6,10,14]. In the infected environment, greater disintegration is observed in the alkaline environment, i.e., in pancreatic juice, for all investigated polymers (Figures S14 and S15).

**Figure 10:**
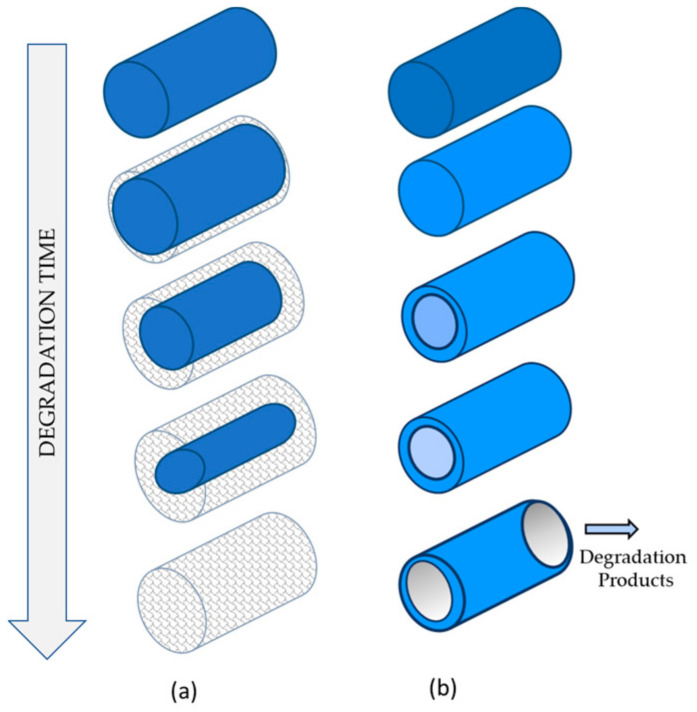
Mechanism of degradation of absorbable polymers: Surface erosion—observed for the Monocryl and the PDS threads (**a**). Bulk degradation—observed for the Vicryl threads (**b**).

Figure 10 illustrates the simplified diagram of the chemical and physical degradation observed for the polymers under study.

### 2.6. Thermal Stability of PDS Surgical Threads

**Figure 11:**
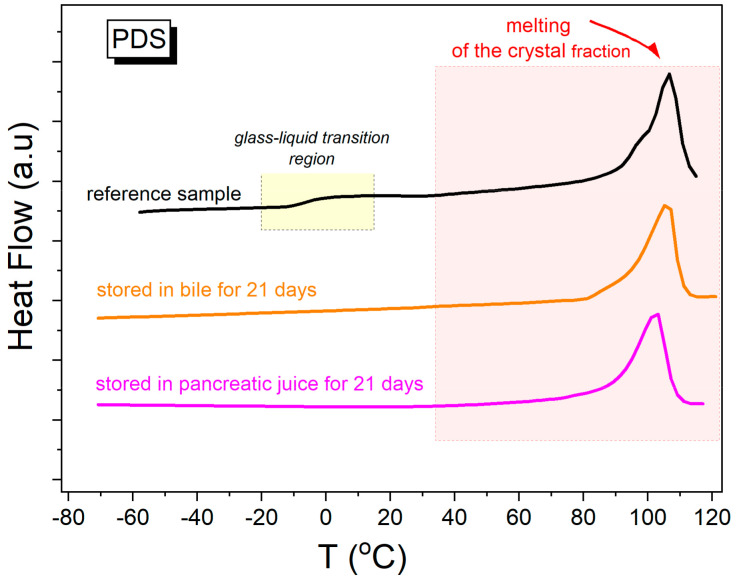
DSC thermograms of fresh surgical threads made from PDS and stored in bile and pancreatic juice for 21 days in a contaminated environment.

The degree of crystallinity is a significant factor influencing the dynamics of degradation. As evidenced by the observations of the bands for alkyl chains (1415, 975), the most significant increase in crystalline areas was observed for the PDS threads. PDS exhibited the lowest susceptibility to hydrolytic degradation and demonstrated the best strength properties. Thermal stability tests were conducted on this polymer. Differential scanning calorimetry (DSC) was employed to compare the thermal properties of fresh polydioxanone (PDS) surgical threads to those of stored threads for 21 days in the pancreatic and bile juices. The obtained DSC thermograms of the examined materials, recorded during heating at a rate of 10 K/min, are presented in Figure 11. The initial (fresh) thread is a semi-crystalline material containing both amorphous and crystalline fractions. At low temperatures, a distinct glass transition (T_g_ = −7 °C) can be identified, while the onset of melting of the crystalline fraction is observed at a temperature of Tm = 90 °C. The melting enthalpy effect is complex, suggesting the presence of several polymorphic forms of the crystalline fraction that differ in Tm values. The total melting enthalpy of the reference sample is ΔH_m_ = 55 J/g.

The storage of surgical threads for more than 21 days in gastric and pancreatic juices resulted in a significant alteration of the thread structure. After 21 days of storage in pancreatic juice, the PDS threads exhibited complete crystallization. In the thermogram obtained during the heating of the sample, the glass transition was no longer identifiable, and only an endothermic process associated with crystal melting was observed (T_m_ = 90 °C, ΔH_m_ = 120 J/g). The same conclusion can be drawn from the DSC thermogram for the PDS thread, which, after 21 days of storage in pancreatic juice, also became fully crystalline. In the thermogram obtained during the heating of this sample, only a broad endothermic process associated with crystal melting was identified (T_m_ = 90 °C, ΔH_m_ = 125 J/g). The significantly higher melting enthalpy values determined for materials degraded in gastric and pancreatic juices indicate a greater degree of crystallinity in these samples compared with the initial one. Therefore, the structural change observed in the calorimetric studies correlates with the change in the mechanical properties of fresh threads and those degraded in bile and pancreatic juices.

## 3. Materials and Methods

### 3.1. Materials

**Figure 12:**
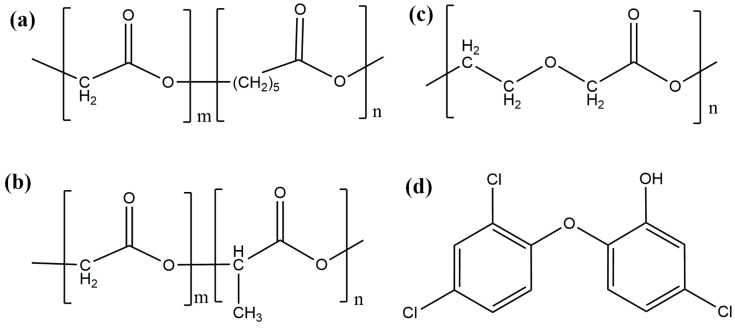
Chemical structure of the polymers from which surgical threads are made. (**a**) Poly(glycolide/ε-caprolactone) Copolymer or Poliglecaprone 25. Suture trade name is Monocryl. (**b**) Poly(glycolide/L-lactide) Copolymer or Polyglactin 910 (m = 90, n = 10). Suture trade name is Vicryl. (**c**) Poly-p-dioxanone. Suture trade name is PDS II. (**d**) Antibacterial coating made of triclosan.

Three types of absorbable threads (PDS, Vicryl, Monocryl), developed by Ethicon, as well as their analogs with antibacterial triclosan coating (PDS plus, Vicryl Plus, Monocryl Plus) were used in the experiment. The inclusion of antimicrobial agents, such as triclosan, is used to confer antimicrobial activity. Figure 12 shows the chemical structure of the absorbable surgical threads with and without antibacterial coating. Triclosan is a chlorinated, aromatic chemical compound that has functional groups characteristic of ethers and phenols, which determine its antimicrobial activity [72,73]. Triclosan is a fungicide and bacteriostatic agent. It has been used as an antibacterial coating for surgical threads.

Surgical Thread Preparation Methodology

Surgical threads with a thickness of 3-0 (according to surgical standards) were immersed in five different environments: (1) sterile pancreatic juice, (2) sterile bile, (3) infected pancreatic juice, (4) infected bile, (5) physiological saline solution (0.9% NaCl)—used as a reference. The biological material was obtained from patients treated at the Department and Clinic of Gastrointestinal Surgery of the Silesian Medical University and at the Department of General, Endocrinological, and Oncological Surgery of the Multispecialty Hospital in Jaworzno. Procedures for preparing the biological material were described in detail in the previous papers [37]. The sterility of the material was confirmed through bacteriological examinations (microscopy and culturing). After collection and distribution into sample tubes, the biological material was frozen in a laboratory freezer ZLN T-200 (POL-EKO) at −20 °C. Immediately after collection, the pH of pancreatic juice was measured using a laboratory pH meter (Piccolo HI 98111, Hanna Instruments, Woonsocket, RI, USA). The pH of pancreatic juice after thawing was 7.7, while that of bile was 6.8. The levels of pancreatic enzymes (amylase and lipase) were determined before freezing and after thawing. The enzymatic activity of the main pancreatic enzymes was assessed by comparing the levels of lipase and amylase before and after thawing. The required amount of pancreatic juice and bile for the planned study was 336 samples of each fluid, as biological materials were exchanged every 24 h. After thawing, bacteriological tests were performed on the fluid in each sample tube. If bacteria were detected in the biological material, the sample tube would be excluded from the study. Contaminated material was obtained by infecting sterile pancreatic juice and sterile bile with strains of bacteria: (1) *Escherichia coli*, (2) *Klebsiella* spp., and (3) *Enterococcus faecalis* (bacteria most commonly found in biliary and pancreatic infections). Threads were immersed in biological material and incubated in sterile sample tubes in a laboratory incubator CL 53 (POL EKO, Wodzislaw Slaski, Poland) at a temperature of +37 °C. The threads were removed from the designated environment at specified time intervals (after 7, 14, 21, and 28 days) and subjected to strength and spectroscopic studies. The sections tested were 20 cm long.

### 3.2. Methods

#### 3.2.1. Fourier-Transform Infrared Spectroscopy (FTIR)

Infrared spectra were recorded on an Agilent Cary 670 spectrometer in the reflectance mode. Reflective infrared spectra were measured using the attenuated total reflection (ATR) technique. The spectrometer was equipped with a DTGS detector and a KBr beam splitter. All spectra in the wavelength range of 400 cm^−1^–4000 cm^−1^ were recorded with a spectral resolution of 2 cm^−1^ and were averaged over 32 scans. The study of structural changes in the material under the influence of the environment was conducted after 0, 7, 14, 21, and 28 days from the immersion of surgical threads in the appropriate media under sterile and contaminated conditions. The absorbance components were determined as the area bounded by the contour of the respective band using the Bio-Rad Win-IR Pro software version 2.96e.

#### 3.2.2. Principal Component Analysis (PCA)

FTIR spectra were pre-processed using second-derivative analysis with a 15-point Savitzky–Golay smoothing algorithm [74]. This pre-processing procedure was rigorously tested to determine the optimal parameters for effectively eliminating optical and scattering artifacts from the spectra while preserving the integrity of the chemical signal [75]. Once satisfactory outcomes were achieved, the data were restricted to the fingerprint range of 950–1800 cm^−1^, a critical region for analyzing polymer structural characteristics. All post-processing analyses were conducted using the Win-IR Pro Bio-Rad software (Bio-Rad Laboratories, Cambridge, MA, USA), which ensured precise handling of spectral data. The processed spectra were subsequently exported to the OriginPro 2023 software (OriginLab Corporation, Northampton, MA, USA), where they underwent additional pre-processing using vector normalization. This approach allowed for the preparation of the data for multivariate analysis [76]. Principal component analysis (PCA) was employed to identify both similarities and differences in the chemical composition of the polymers. This analysis also facilitated a qualitative examination of the impact of environmental factors and time intervals on the molecular structure of the polymers. The PCA revealed distinct patterns of behavior among the different polymers, highlighting the impact of various environmental conditions on their stability and degradation over time.

#### 3.2.3. Density Functional Theory (DFT) Calculations

To conduct an in-depth analysis of the experimental spectra, theoretical FTIR spectra for isolated molecules were calculated using density functional theory (DFT). In this work, electronic structure calculations of polymers were performed using the Gaussian09 program (version E.01) [58]. Molecular structures, harmonic force constants, and absolute FTIR intensities were determined using DFT with Becke’s three-parameter exchange functional in combination with the Lee, Yang, and Parr correlation functional B3-LYP method with the basis set 6-311(d,p) [59]. The results were visualized using the GaussView 5.0.8 software. Discrete spectral lines were broadened using Gaussian functions with a half-width of 7 cm^−1^. Then, the frequencies were scaled using a scaling factor determined for the applied basis, which was 0.967 ± 0.021, according to the Computational Chemistry Comparison and Benchmark Database. Band intensities were divided by the intensity of the highest band in the theoretical spectrum for each molecule.

#### 3.2.4. Mechanical Strength Testing

Strength measurements of surgical threads were performed on an INSTRON 4469 strength testing machine (Instron ^®^, Norwood, MA, USA). The measurement range of the test head was 5 kN, with a measurement accuracy of 0.1 N and a crosshead speed (strain) of 20 mm/min. The resistance of the material to environmental conditions was tested after 0, 7, 14, 21, and 28 days of surgical thread immersion. Tensile strength was calculated as the ratio of the measured tensile force obtained during a static tensile test related to the original cross-sectional area of the threads. Day “0” represented the date of measurement of the suture taken directly from the packaging (initial state). Details of the strength testing procedure and the analysis of results with statistical analysis have been described in our previous work [36].

#### 3.2.5. Differential Scanning Calorimetry (DSC)

For the PDS II material, which underwent the slowest biodegradation within a month, additional thermal stability tests were conducted. A reference thread made of PDS II and stored for 21 days, both in sterile and contaminated environments, was subjected to calorimetric measurements using a Mettler-Toledo DSC apparatus equipped with an HSS8 ceramic sensor (heat-flow sensor with 120 thermocouples) and a liquid nitrogen cooling accessory. Thermograms were collected during the cooling and heating in the temperature range of −80 °C to 120 °C. The cooling and heating rates were 10 K/min. Calorimetric measurements were conducted in a nitrogen atmosphere at a flow rate of 60 mL/min.

#### 3.2.6. Scanning Electron Microscopy (SEM)

The Hitachi S-3400N microscope (Hitachi Ltd., Japan), capable of magnification from 5× to 100,000×, was utilized. The device was equipped with X-ray spectrometers—Thermo Noran EDS and Thermo MagnaRay WDS (EDS—energy-dispersive spectroscopy; WDS—wavelength-dispersive spectroscopy)—as well as a backscattered electron diffraction detector (EBSD). Micrographs of the surgical threads were obtained from backscattered electrons (BSEs). All observations and image acquisitions were conducted at an accelerating voltage of 15 kV.

## 4. Conclusions

This study investigated the degradation mechanisms and factors affecting synthetic polymers used in surgical threads. Critical parameters such as tensile strength, degradation rate, and material stability are crucial for selecting appropriate materials for surgical use.

Infrared spectroscopy, in combination with multivariate statistical analysis, including the PCA approach, was employed to analyze structural changes in polymers exposed to body fluids over time. The analysis focused on the characteristic vibrations of ester (COO⁻) and ether (COC) groups, which are most susceptible to hydrolytic degradation. Significant decreases in ester group intensity, particularly in Vicryl sutures, were linked to ester bond cleavage. The emergence of a new band at around 1710 cm^−^^1^ in Vicryl suggested the formation of α-hydroxy acids. An increase in crystallinity, especially in PDS sutures, was confirmed by DSC studies and correlated with spectroscopic data.

The study revealed that polymer hydrolysis is a complex process influenced by crystallinity. Two distinct degradation mechanisms were identified: water absorption leading to polymer chain plasticization in Monocryl and direct ester bond degradation initiating in the amorphous phase in PDS and Vicryl threads. SEM analysis showed surface erosion in pancreatic juice and volumetric erosion in bile, with Vicryl threads exhibiting the most pronounced degradation.

Coating surgical threads with antifungal and antibacterial materials was found to slow down degradation, maintaining polymer integrity and prolonging their support in wounds. PDS threads demonstrated optimal properties for environments exposed to pancreatic juice and bile, aligning with previous strength tests.

In conclusion, this study provides valuable insights for selecting absorbable surgical threads for challenging environments. It offers guidance for designing future polymer blends or modifications to enhance controlled degradation in clinical applications.

## Figures and Tables

**Table 1:** Vibrational frequencies/wavenumber (ν), relative intensity of the bands (I_R_), and assignments of the prominent bands for all polymeric threads.

EXP						Assignments
Monocryl		Vicryl		PDS		
ν (cm^−1^)	I_R_ (a.u.)	ν (cm^−1^)	I_R_ (a.u.).	ν (cm^−1^)	I_R_ (a.u.)	
515	w	535 563	w	580	w	γ C=O, out of plane def.
628	m	665 685	m	703	m	β C=O, in-plane def. (COO) +νCCC
720	m,br	760	m, br	723	m	δCH_2_ rocking
807 850	m	808 850	m	850 873 932	m	ν_s_ COC
955	m	--	--	930	m	ν_as_ C-O-C + νCCC (skeletal)
975	m	975	m	--	--	νCCC (skeletal)
--	--	1047	m, sh	1050	s	ν_as_ O-C-C
1087	s	1090	s	1070	s	ν_as_ O-C-C in glycolide seg.
1150	vs	1160 1180	vs	1120 1202	s s,sh	ν_as_ C-C(O)-O in ester
-- 1742	-- vs	1720 1745	s, sh vs	1734 1746	s, sh vs	νC=O
2850 2870	vw	2849	vw	2851 2882	m	ν_s_CH_2_ ν_as_CH_2_
2916 2957	vw	2917 2957	vw	2958 2989	m	ν_s_CH_3_ ν_as_CH_3_

Key: s—symmetrical; as—asymmetric; Al —alkyl chain; νCCC—skeletal; ν—stretching; γ—deforming out of plane; γ—deforming in plane; δ—deforming; vs—very strong; s—strong; m—medium; w—weak; vw—very weak; sh—shoulder; br—broadband; Ir—relative intensity of the bands.

## Data Availability

Data contained within the article.

## Supplementary Materials

The following supporting information can be downloaded at: https://www.mdpi.com/article/10.3390/ijms252011333/s1.
