# Supplementary material for: The Degradation of Absorbable Surgical Threads in Body Fluids: Insights from Infrared Spectroscopy Studies
Source: Int J Mol Sci. 2024 Oct 21;25(20):11333. doi: 10.3390/ijms252011333 (PMC11508208; doi:10.3390/ijms252011333)
Supplement: Supplementary file 1 [file ijms-25-11333-s001.zip › ijms-3233397-supplementary.pdf]

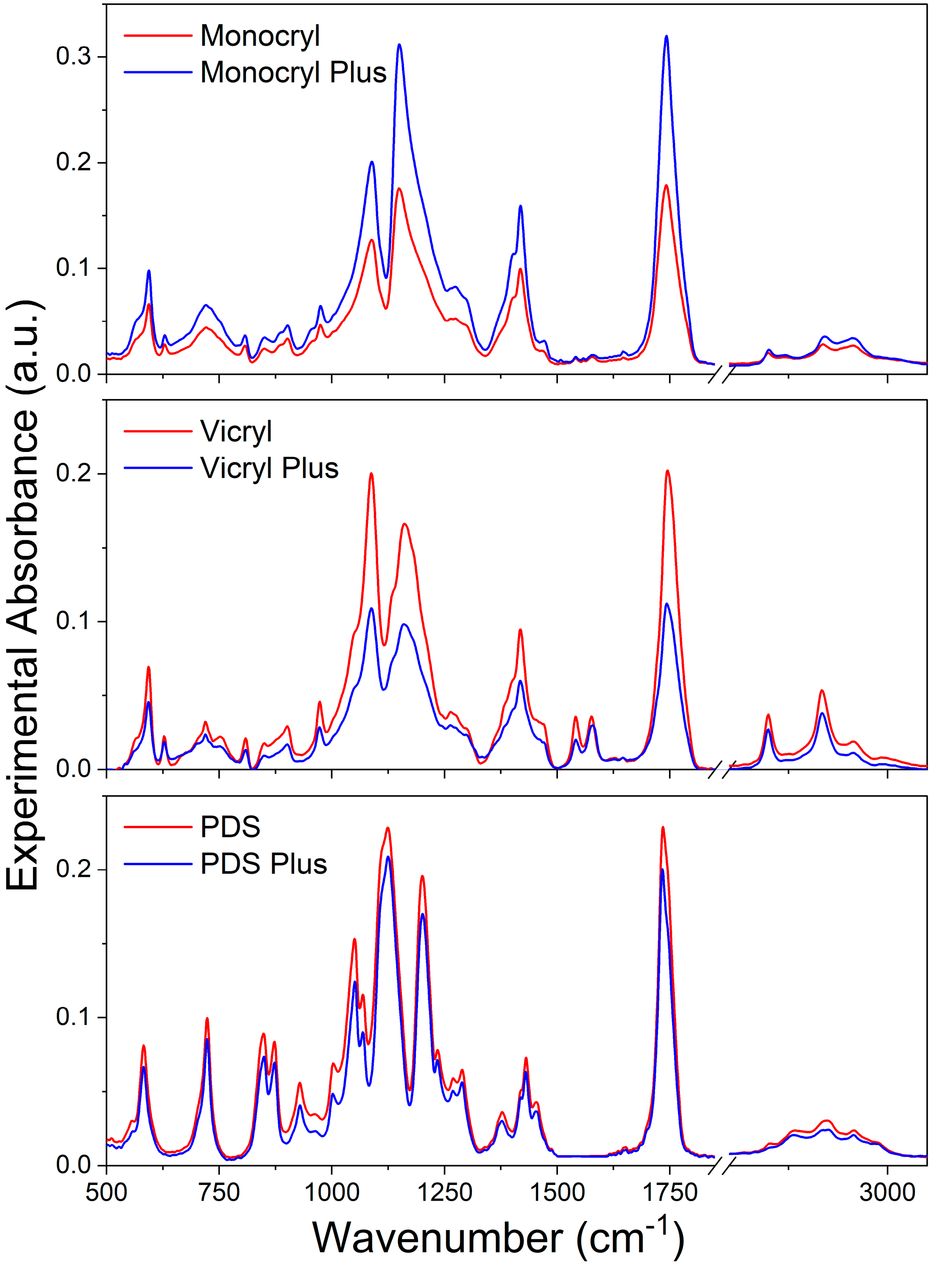

Figure S1. Comparison of FTIR spectra of raw Monocryl and Monocryl Plus threads over the full range of wave numbers (500-3500cm-1).

Description of abbreviations and symbols contained in tables S1-S3.

Simulated FTIR Spectra:

Band spectrum: ν - vibration frequency expressed in wave numbers (cm^{-1}) scaled with a scaling factor (0.976); I_R - Relative intensity of the bands,

Experimental FTIR Spectra:

ν - wavenumber (cm^{-1}), I_R - Relative intensity of the bands

Key of vibration assignment: s – symmetrical, as – asymmetric, ν – stretching, γ – out of plane bending vibration: wagging and twisting, β & δ – in-plane bending: scissoring and rocking vibration, vs – very strong, s – strong, m – medium, w – weak, vw – very weak, sh – shoulder, am – amorphous phase, cr – crystalline phase, ls - long sequence.

Table S1. Theoretical and experimental frequencies, relative intensity and approximate band assignments for Monocryl and Monocryl Plus.

EXP				SYM		ASSIGNMENTS
MONOCRYL		MONOCRYL plus				
ν (cm^{-1})	I_R (a.u.)	ν (cm^{-1})	I_R (a.u.)	ν (cm^{-1})	I_R (a.u.)	
515	w	515	w	513	w	γ C=O, out of plane def.
537<br>564	w<br>w	540<br>560	w<br>w	545<br>582	w<br>w	βC=O, in plane def.
590<br>628	m<br>w, cr	594<br>630	m<br>w, cr	602<br>636<br>660	vvw	δCCC def. + βCOO def.
720	w, br, am	720	w, br, am	729<br>755	vvw	δCH_2 rocking
807<br>850	w, cr<br>w, am	811<br>850	w, cr<br>w, am	805<br>822	w<br>w	ν_s COC + δ_{as}^{-} CH_3 (rocking)
884<br>902	w, sh, am<br>w, cr	885<br>900	w, sh, am<br>w, cr	918	w	δ_{as}^{-} CH_3 (rocking)
955	w	955	w	950	w	ν_{as} O-C-C in ε-Caprolactone segment
975	w, cr	974	w, cr	970	w	νCCC (skeletal) + ν_{as} O-C-C
1087	vs, ls	1085	s, ls	1083	vs	ν_{as} O-C-C in Glycolide seg.
1150	vs<br>am + cr	1150	vs<br>am + cr	1163<br>1180<br>1195<br>1219	vs<br>m, sh<br>m, sh<br>w	ν_{as} C-C(O)-O<br>+ γ_s CH_2 (wagging)

1385	m, sh, am	1389	m, sh, am	1390	m	δ_{as}^{+} CH_3 (umbrella) + γCH (wag-ging)
1417	s, cr	1419	s, cr	1435	m	
1540	vw	1542	vw	1532	vw	β_s CH_2 (scisoring)
1580		1580		1560	vw	
1742	vs cr + am	1742	vs cr + am	1744	vw	νC=O
2850	w	2850	w	--	vw	$\nu_s CH_2$
2870		2870		2870		$\nu_{as} CH_2$
2916	w	2920	w	2933	vw	$\nu_s CH_3$
2957		2957		2946		$\nu_{as} CH_3$

Table S2. Theoretical and experimental frequencies, relative intensity and approximate band assignments for Vicryl and Vicryl Plus.

**EXP**				**SYM**		**ASSIGNMENTS**
**VICRYL**		**VICRYL plus**				
**ν (cm^{-1})**	**I_R (a.u.)**	**ν (cm^{-1})**	**I_R (a.u.)**	**ν (cm^{-1})**	**I_R (a.u.)**	
535	m, sh	535	m	540	vvw	γ C=O
563	m	594	m	565		
665	w	667	w	670	vvw	βC=O + νCCC skeletal
684	w	680	w			
760	w, am	762	w, am	756	vvw	δ CH_2 (rocking) + βC=O
808	w, cr	810	w, cr	812	w	ν_s COC
850	w, am	850	w, am	840		ν_s COC + δ_{as}^{-} CH_3 (rocking)
900	w, cr	905	w, cr	866	w, sh	δ_{as}^{-} CH_3 (rocking)
				900	w	
975	m, cr	974	m, cr	980	m	νCCC skeletal + ν_{as} O-C(CH_3) in L-Lactide segment
1047	m, sh	1047	m, sh	1028	vw	ν_{as} O-C(CH_3) in L-Lactide segment + νCCC skeletal
				1052		
1090	vs, ls	1088	s, ls	1095	vs	ν_{as} O-C-C in Glycolide seg.
1133	s, sh, am	1132	s, sh, am			ν_{as} C-C(O)-O in L-Lactide segment
1161	vs, cr	1161	vs, cr	1157	vs	ν_{as} C-C(O)-O in Glycolide seg.
1183	w, sh, cr	--	--			
1270	w, sh	1270	w, sh	1276	vw	γ_{as} CH_2 (twisting)
1398	m, sh, am	1398	w, sh, am	1406	w, sh	δ_{as}^{+} CH_3 (umbrella)
1420	m, cr	1420	m, cr	1417	m	
1720	s, sh	1720	m, sh	1745	vw	νC=O,
1745	vs	1742	s			
2849	w	2850	w	2852	vw	$\nu_s CH_2$
--	--	--	--	2868		$\nu_{as} CH_2$

2917	w	2917	w	2910	vw	$\nu_s CH_3$
2959	w	2960	w	2973		$\nu_{as} CH_3$

Table S3. Theoretical and experimental frequencies, relative intensity and approximate band assignments for PDS and PDS Plus.

**EXP**				**SYM**		**ASSIGNMENTS**
**PDS**		**PDS plus**				
**ν (cm^{-1})**	**I_R (a.u.)**	**ν (cm^{-1})**	**I_R (a.u.)**	**ν (cm-1)**	**I_R (a.u.)**	
550	w, sh	557	w	545	w	δCCC def
582	w	579	w	570	w	γ C=O, out of plane def.
703	m, sh	703	m, sh	714	w	βC=O, in plane def. (COO) +νCCC
723	m, am	723	m, am	737	vw	δCH_2 rocking
844	m, am	846, am	m	846	w	δ_{as}^{-} CH_3 (rocking)
872	m, am	873, am	m	877	w	ν_s COC
928	m, cr	930	m, cr	922	w	ν_{as} C-O-C
1002	m, sh	1000	m, sh	1020	w	nCCC (skeletal)
1050	s, am	1050	s, am	--	--	ν_{as} C-O-C
1070	s, ls	1070	s, ls	--	--	ν_{as} O-C-C in Glycolide seg.
1108	s, sh	1110	s, sh	1093	s, sh	
1126	vs, am + cr	1125	s, sh am + cr	1130	s	ν_{as} C-C(O)-O (in ester)
1202	s, cr	1201	vs, cr	1160	s, sh	
1237	m, sh	1235	m	1205	w, sh	ν_{as} C-C(O)-O (ethyl acetate)+ $\gamma_s CH_2$
1270	m	1267	m	1285	w	$\gamma_s CH_2$
1290		1290		1300		$\gamma_{as} CH_2$
1378	w, am	1365	w, am	1350<br>1360	vw	δ_{as}^{+} CH_3 (umbrella) + $\gamma_s CH_2$
1420	m, cr	1420	m, cr	1390	w, sh	$\gamma_{as} CH_2$
1430	m, am	1430	m, am	1405	m	
				1435		
1455	w, am	1455	w, am	1450	w	$\beta_s CH_2$ (scisoring)
				1475		
1734	vs, am	1733	vs, am	1740	s	νC=O
1746	vs, sh, am + cr	1745	vs, sh, am + cr			
2851	vw	2850	vw	2873	m	$\nu_s CH_2$
2882		2881		295		$\nu_{as} CH_2$
2922	vw	2918	vw	2960	m	νCH

		2925				
2958	vw	2957	vw	2990	m	$\nu_s CH_3$
2989		2988		3020		$\nu_{as} CH_3$

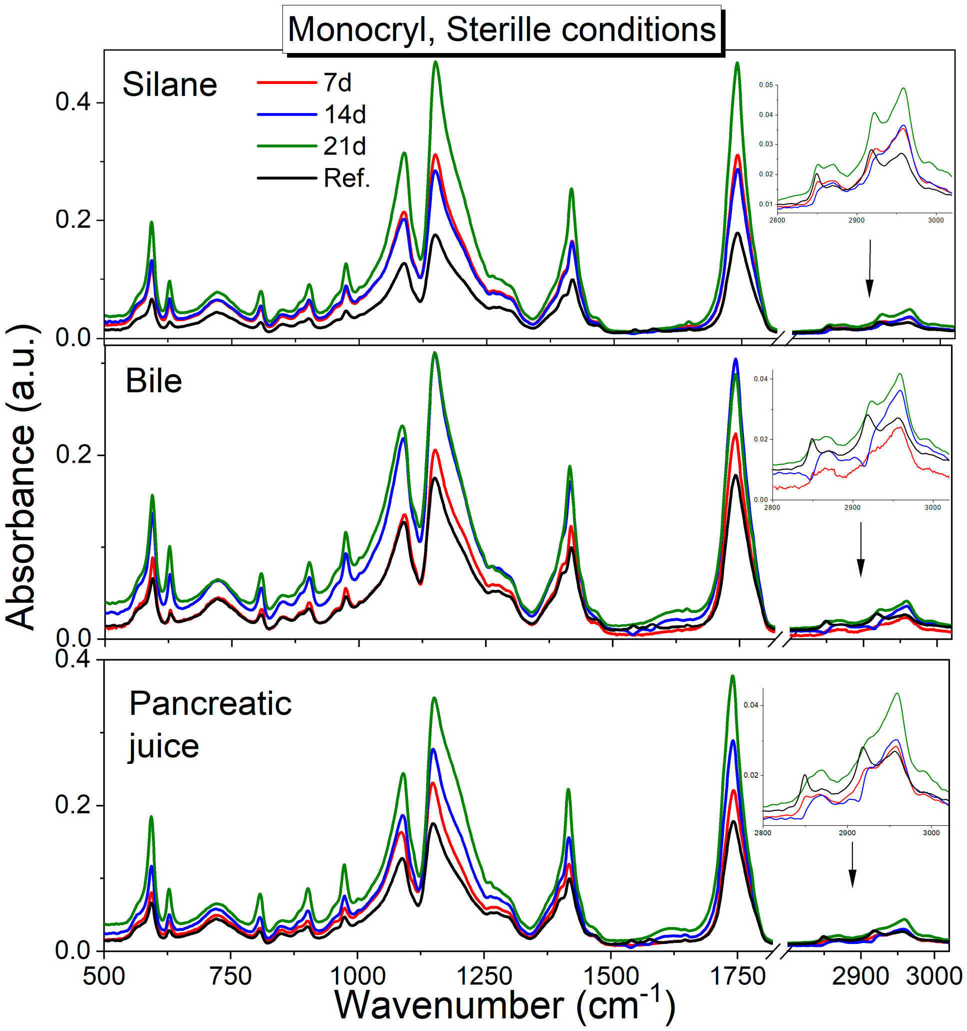

Figure S2. FTIR spectra in the 500-3500 cm^{-1} range for the Monocryl surgical suture immersed in sterile environment in various body fluids for 7, 14 and 21 days. (a) saline , (b) bile, (c) pancreatic juice.

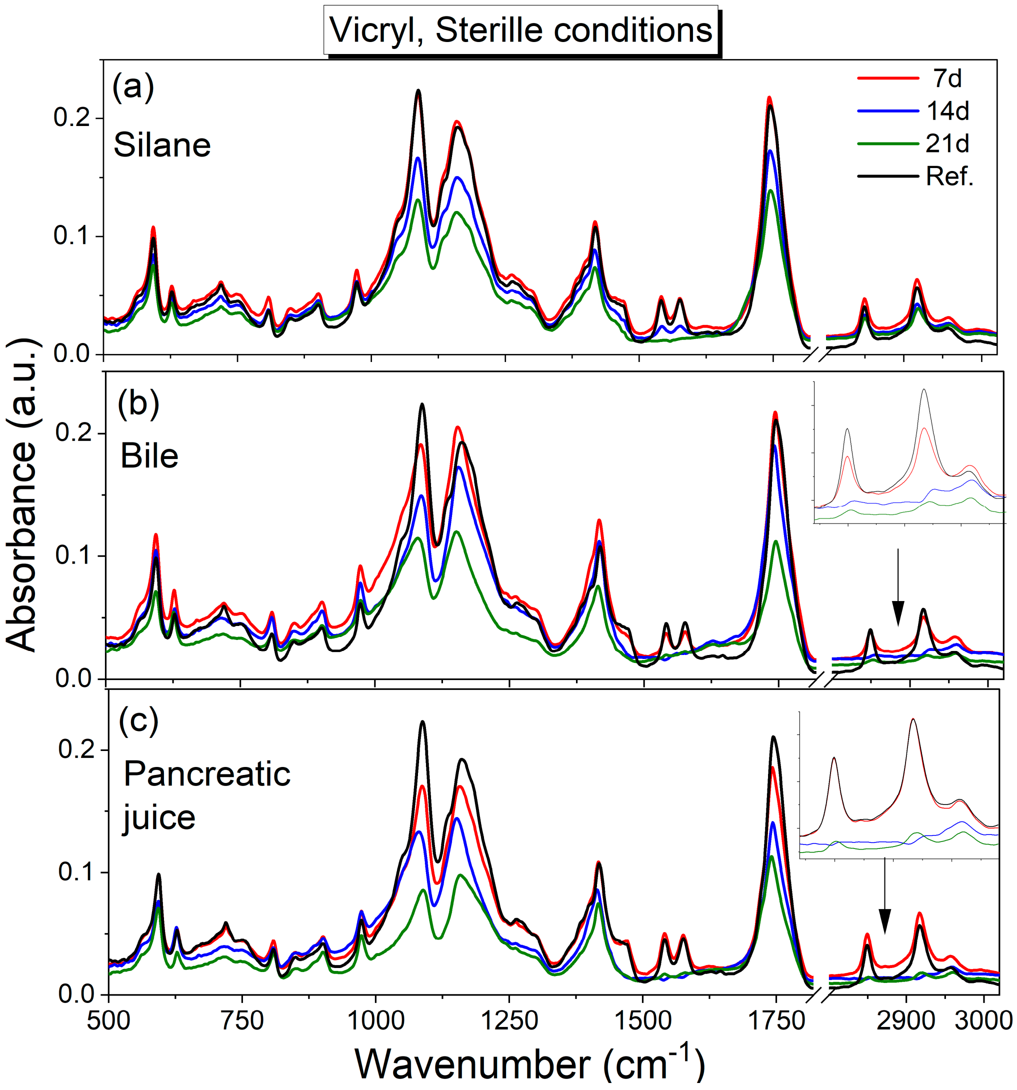

Figure S3. FTIR spectra in the 500-3500 cm^{-1} range for the Vicryl surgical suture immersed in sterile environment in various body fluids for 7, 14 and 21 days. (a) saline , (b) bile, (c) pancreatic juice.

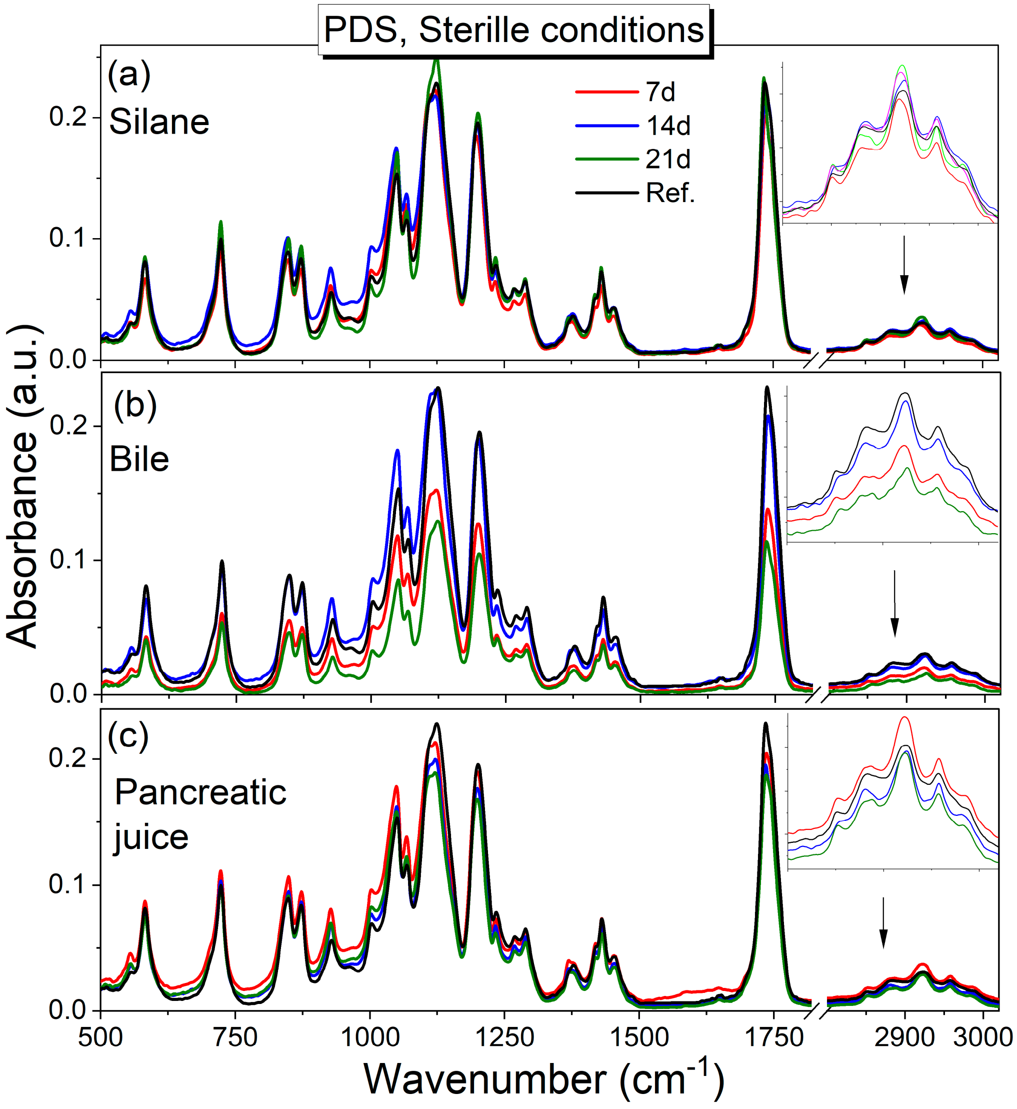

Figure S4. FTIR spectra in the 500-3500 cm^{-1} range for the PDS surgical suture immersed in sterile environment in various body fluids for 7, 14 and 21 days. (a) saline , (b) bile, (c) pancreatic juice.

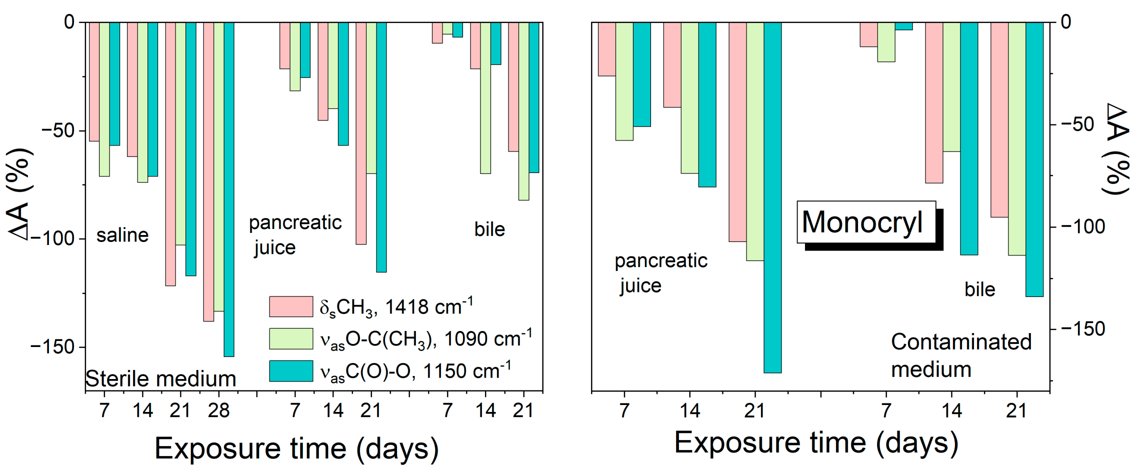

(**a**) (**b**)

Figure S5. Average percentage increase in integral absorbance for degradation-sensitive bands calculated with reference absorbance before degradation for Monocryl suture. (a) Sterile and (b) contaminated media. $\beta_s CH_2$ - deformational vibrations of the methylene group (scissoring) in a ε-Caprolactone segment in the crystal phase (1418 cm^{-1}), ν_{as} O-C$_{Al}$ - asymmetric stretching vibration of the group O-C-C in ε-Caprolactone segment (1090 cm^{-1}),′ ν_{as} C-C(O)-O asymmetric stretching vibration of the ester group.

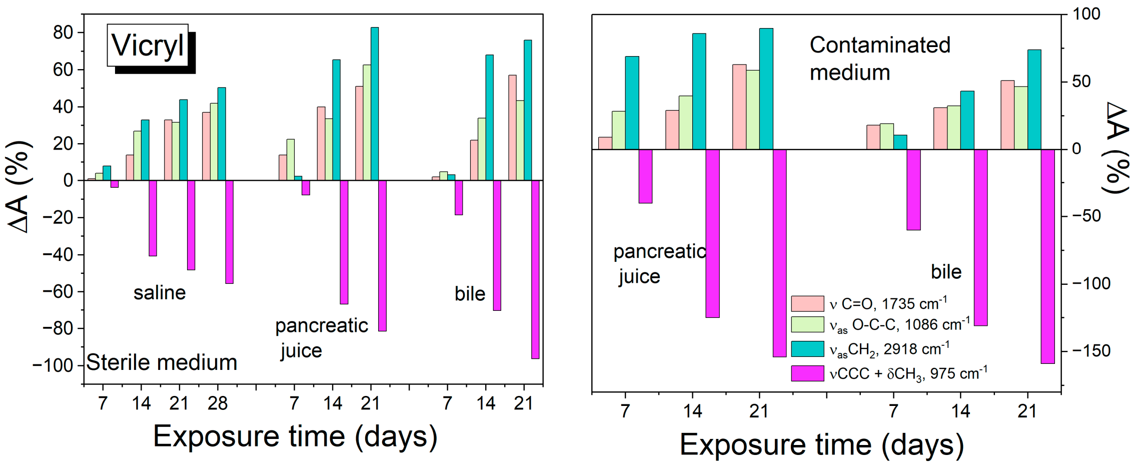

(**a**) (**b**)

Figure S6. Average percentage change in integral absorbance for degradation-sensitive bands calculated with reference absorbance before degradation for Vicryl suture. (a) Sterile and (b) contaminated media. ν C=O – stretching vibration of carbonyl group, ν_{as} O-C-C - asymmetric stretching vibration in Glycolide seg.(1086 cm^{-1}), νCCC – skeletal vibration (975cm^{-1}).

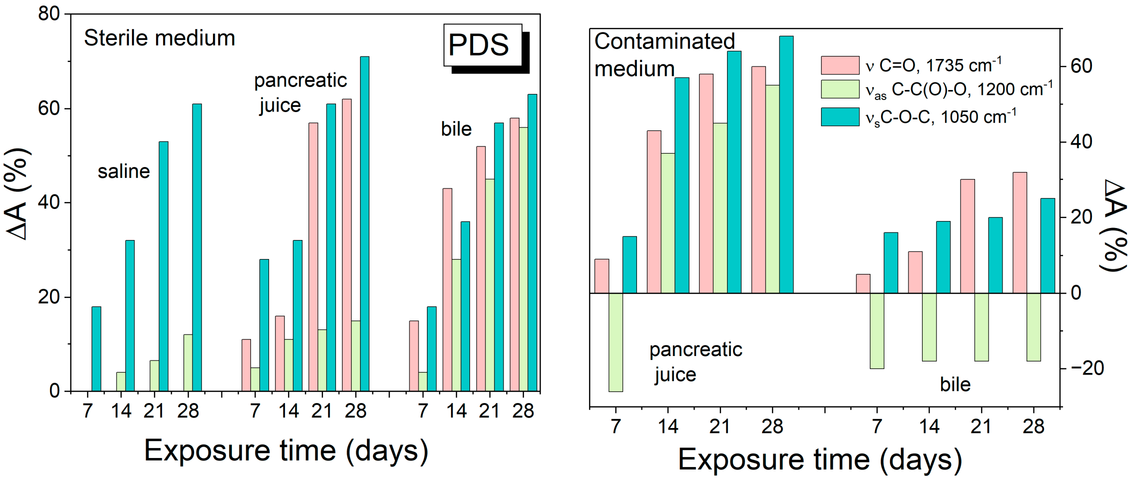

(**a**) (**b**)

Figure S7. Average percentage decrease in integral absorbance for degradation-sensitive bands calculated with reference absorbance before degradation for PDS suture. (a) Sterile and (b) contaminated media. ν C=O – stretching vibration of carbonyl group, ν_{as} C-O-C - asymmetric stretching vibration in ether group (1050 cm^{-1}), ν_{as} C-C(O)-O asymmetric stretching vibration of the ester group.

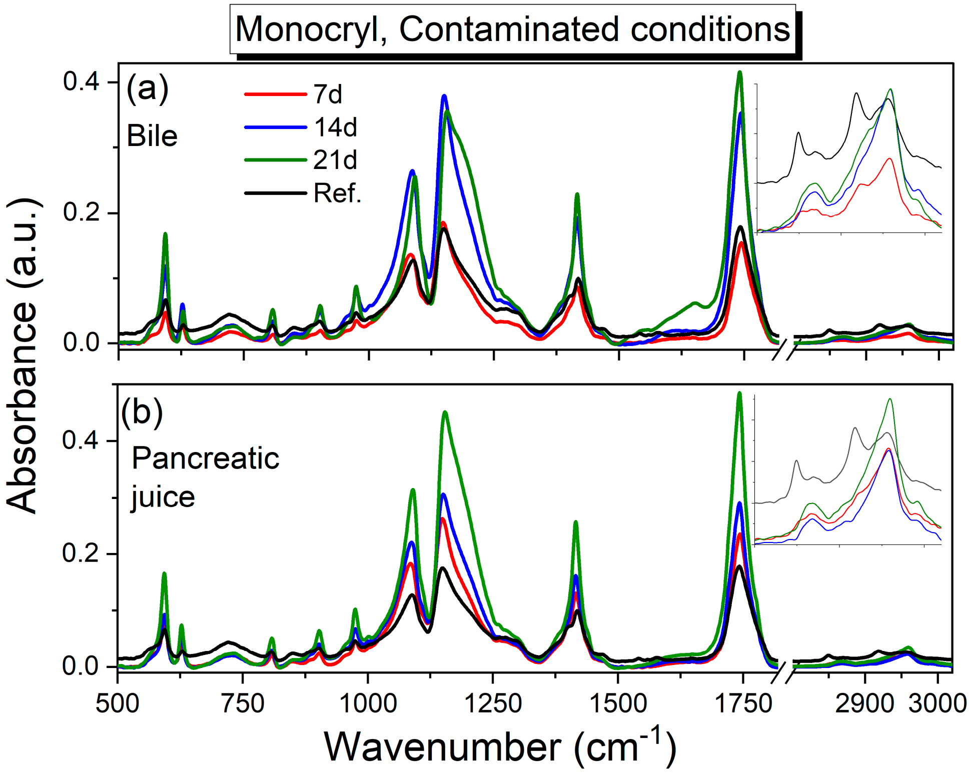

Figure S8. FT-IR spectra in the 500-3500 cm-1 range for the Monocryl surgical suture immersed in contaminated environment in various body fluids for 7, 14 and 21 days. (a) bile and (b) pancreatic juice.

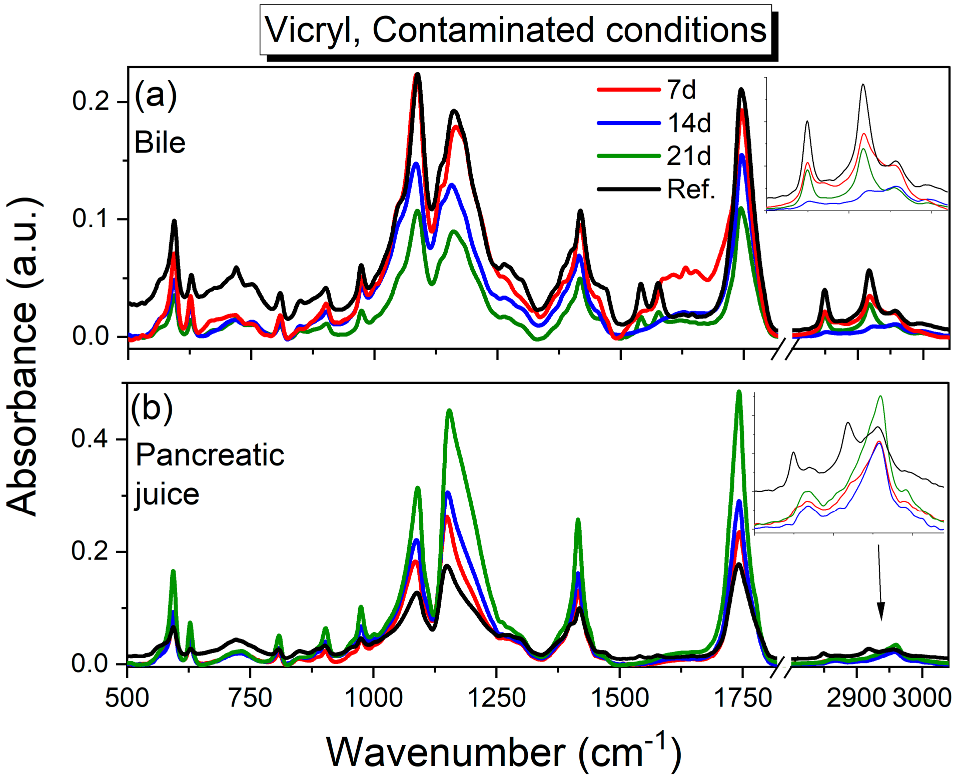

Figure S9. FT-IR spectra in the 500-3500 cm^{-1} range for the Vicryl surgical suture immersed in contaminated environment in various body fluids for 7, 14 and 21 days. (a) bile and (b) pancreatic juice.

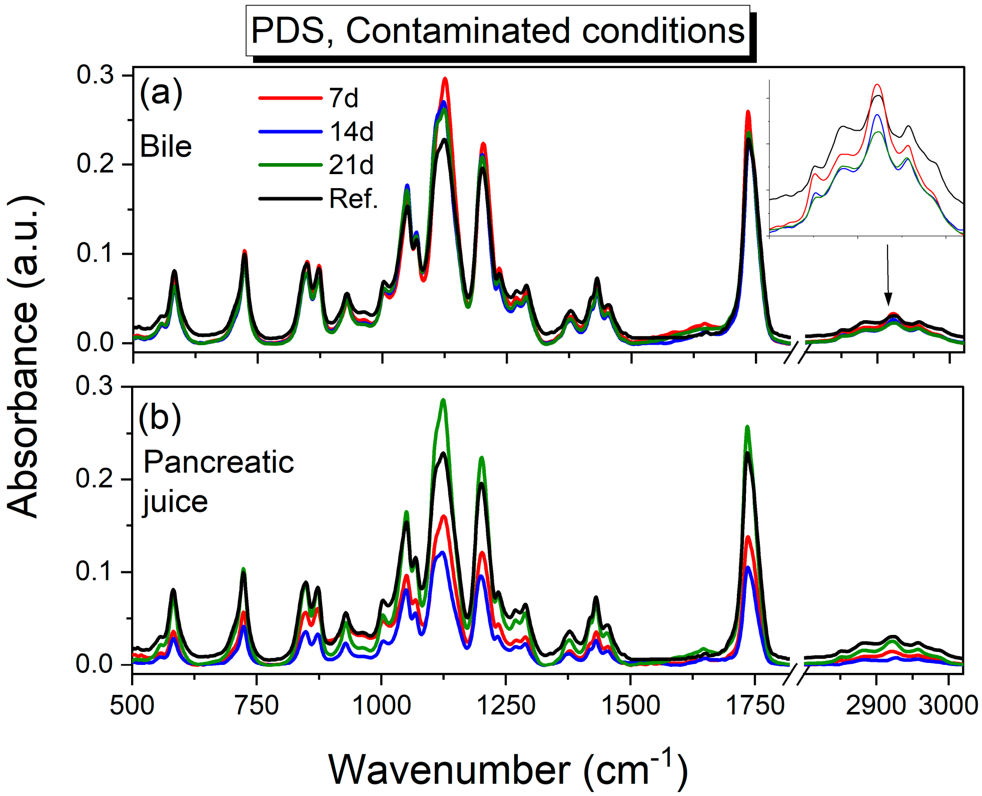

Figure S10. FT-IR spectra in the 500-3500 cm-1 range for the PDS surgical suture immersed in contaminated environment in various body fluids for 7, 14 and 21 days. (a) bile and (b) pancreatic juice.

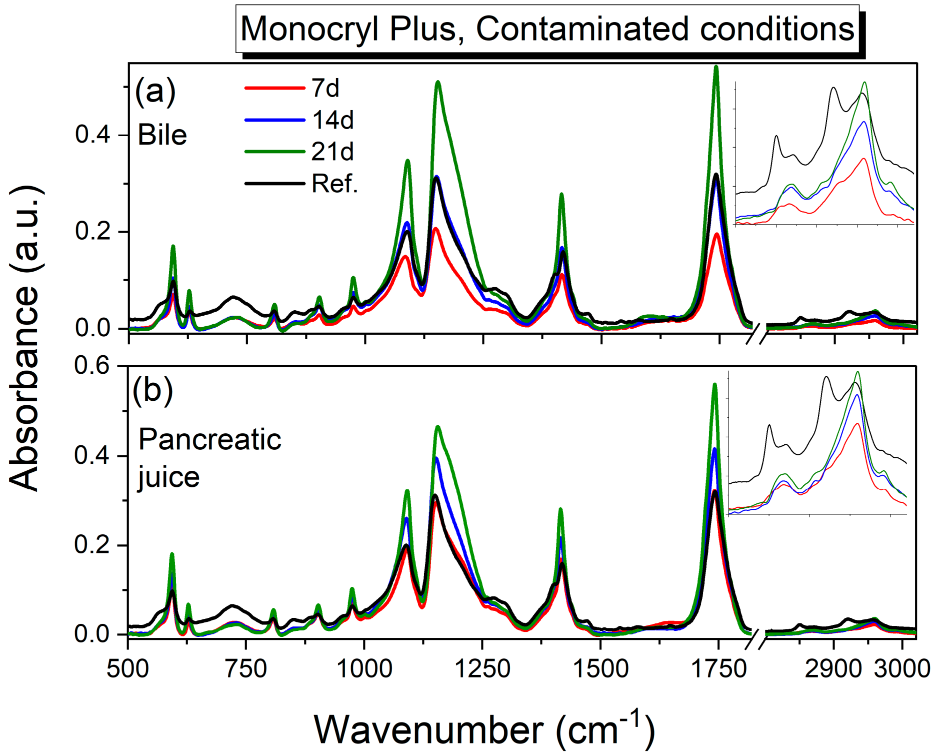

Figure S11. FT-IR spectra in the 500-3500 cm^{-1} range for the Monocryl Plus surgical suture immersed in contaminated environment in various body fluids for 7, 14 and 21 days. (a) bile and (b) pancreatic juice.

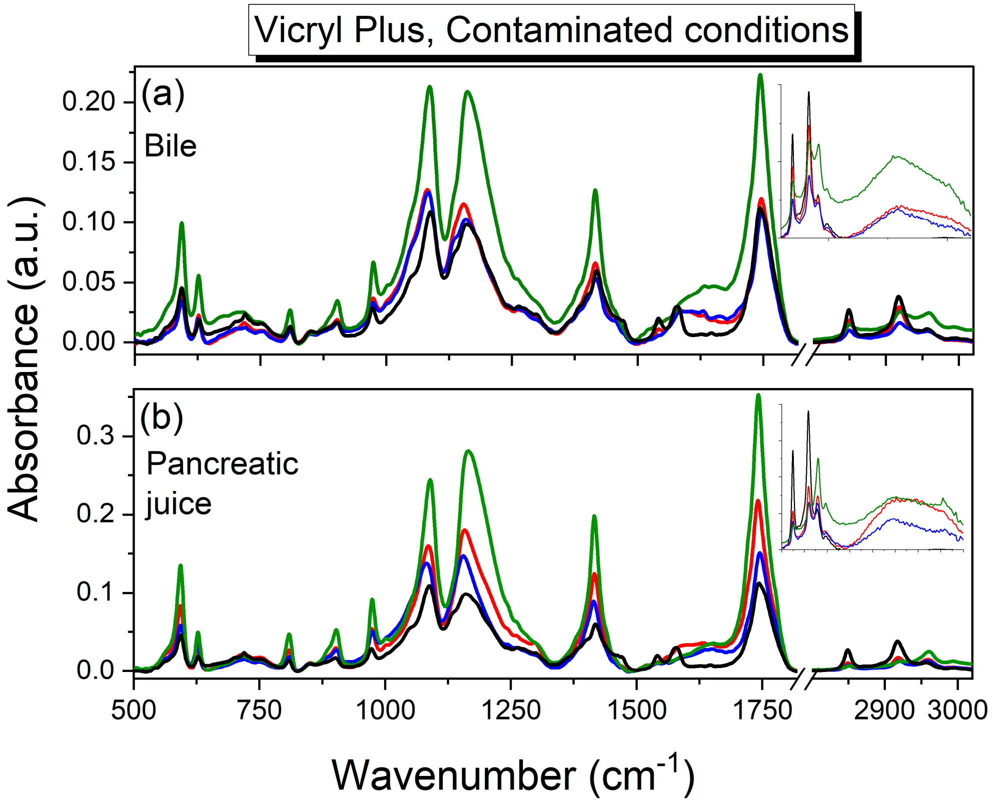

Figure S12. FT-IR spectra in the 500-3500 cm^{-1} range for the Vicryl Plus surgical suture immersed in contaminated environment in various body fluids for 7, 14 and 21 days. (a) bile and (b) pancreatic juice.

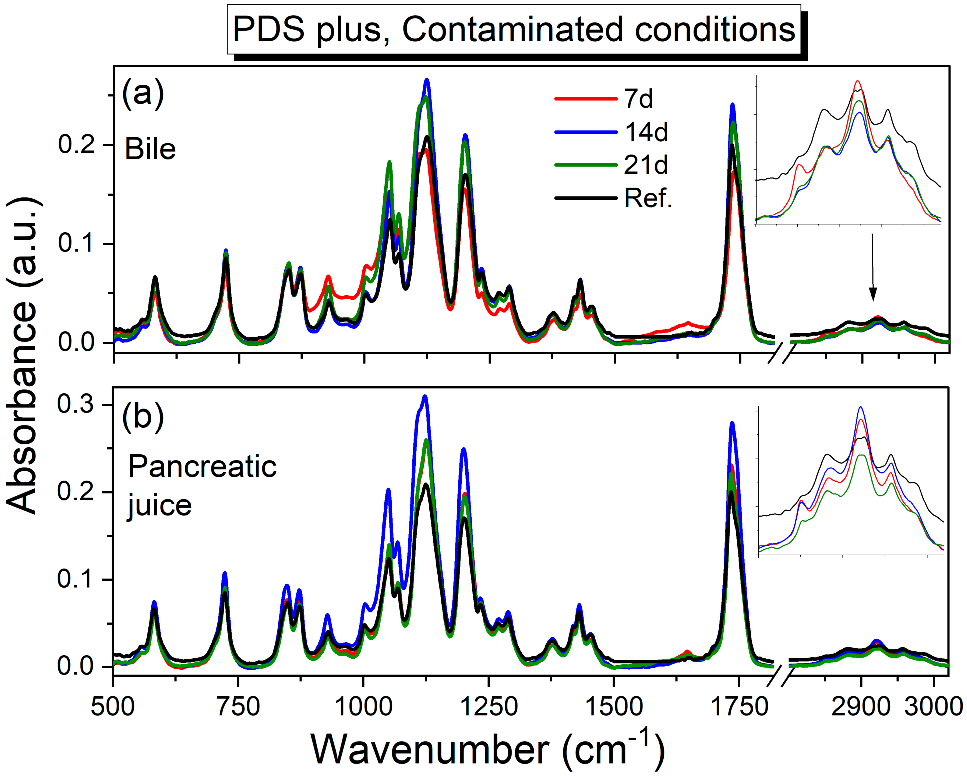

Figure S13. FT-IR spectra in the 500-3500 cm^{-1} range for the PDS Plus surgical suture immersed in contaminated environment in various body fluids for 7, 14 and 21 days. (a) bile and (b) pancreatic juice.

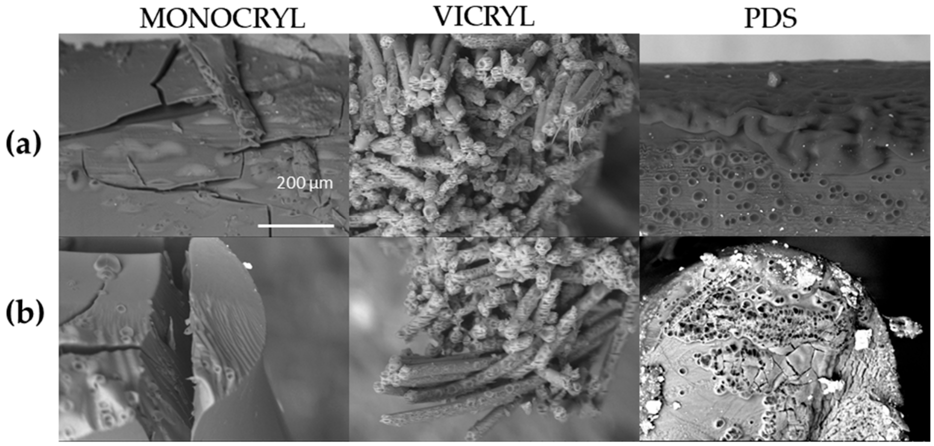

Figure S14. Scanning electron microscope images of uncoated surgical threads immersed for 21 days in in contaminated environment of pancreatic juice. **(a)** view of the side surface of the suture, **(b)** cross-sectional view of the suture.

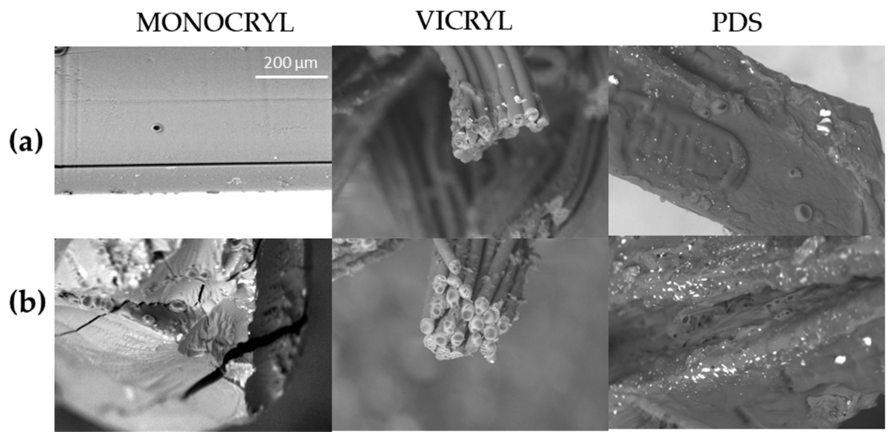

Figure S15. Scanning electron microscope images of uncoated surgical threads immersed for 21 days in in contaminated environment of bile. (a) view of the side surface of the suture, (b) cross-sectional view of the suture.
